# A comprehensive scheme for power management of FC/SC/battery, and solar-roof PV source in electric vehicle systems

**DOI:** 10.1038/s41598-024-79241-1

**Published:** 2024-11-11

**Authors:** Majid Valizadeh, Mahyar Shiri, Amirhosein Khosravi Sarvenoee, N. Gowtham, Kareem M. AboRas

**Affiliations:** 1 Department of Electrical Engineering, Ilam University, 6931647574 Ilam, Iran; 2 Department of Electrical and Electronics Engineering, Manipal Institute of Technology Bengaluru, Manipal Academy of Higher Education, Manipal, Karnataka 576104 India; 3 Department of Electrical Power and Machines, Faculty of Engineering, Alexandria University, Alexandria, 21544 Egypt

**Keywords:** Fuel-cell, PV, High step-up Converter, Evolutionary algorithm, Fractional Order PI-Controller, MPPT, Energy science and technology, Engineering

## Abstract

This paper proposes a new energy management system to combine Fuel Cells (FC) and photovoltaic (PV) panels as primary power sources. Also, battery and Super Capacitor (SC) banks are considered as secondary energy systems. The high power density and the fast dynamics of SCs combined with the high energy density and medium dynamics of batteries would be an ideal combination for FC vehicles. The high step-up DC/DC converter is used along with the implementation of the maximum power point tracking algorithm (MPPT) to adapt the low power of PV panels with the high power of DC-Link. Also, an evolutionary optimization algorithm is proposed along with the FOPI controller to improve the output of the energy management algorithm and control the sources more efficiently. The simulation results are validated for a standard urban driving cycle. It indicates for the case of the sustained presence of a PV source during a typical driving cycle, the source delivers about 55% of its maximum power.

**Supplementary Information:**

The online version contains supplementary material available at 10.1038/s41598-024-79241-1.

## Introduction

By the growth up of urbanization and industrialization of the world, the desire for personal vehicles will have an unprecedented growth that highlights the challenges of global warming due to the massive use of fossil fuels by transportation systems. The number of available energy resources in cars has increased^1^. In recent years, the need for an energy source with higher efficiency and zero (or near zero) pollution regained the consideration of FCs as an essential source in future vehicle engines^2^. Currently, several efforts have been devoted to the study of FC hybrid electric vehicles (FCHEVs), where the main power is supplied by the FC, and a battery source is used as a secondary energy storage element. However, the battery has some issues such as low life cycle, high charge time, and low power density. These restrictions can be alleviated by the use of a SC, which has a high life cycle, fast charging time, and high power density as opposed to the typical battery. A SC can provide the high power required to accelerate the vehicle and efficiently store the energy generated by the regenerative braking. Due to these characteristics, SCs can be used as suitable secondary energy sources alongside batteries^3^. The results of a combination of FCs and SCs show that SCs can be an effective factor in offsetting the lack of hydrogen fuel and low FC response speeds in transient states of vehicle required power^4^. Nomenclatures are shown in Abbreviations section.

The deployment of PV systems on the outer surfaces of electric vehicles (EVs), plug-in hybrids (PHEVs), or FCHEVs can reduce the overall fuel consumption. Also, PV systems are lightweight, silent, maintenance- free, and work continuously even when the vehicle is not operating. In FCHEVs, PV systems can be used as a low power auxiliary system. The main barrier against the utilization of PV system so far was its high price which has been significantly reduced over the past decade. This trend is expected to continue in the future^5^. However, the solar panels have been re-considered by automakers to improve the passenger’s comfort. Vehicles like the 2010Prius, Aptera2, Audi A8, and Mazda 929 have sunroof features with thematic targets^6^.

In general, the operational voltage of hybrid power systems is different from the DC common bus voltage for various power sources and external loads. The power storage sources must have a bi-directional power transmission. Therefore, it is necessary to boost and/or bulk the voltages of different power sources to ensure the DC-bus voltage operates at a stable level.

DC /DC converters can be used to interface these elements in the electric power train by boosting or chopping the voltage levels. Due to the automotive constraints, the power converter structure has to be reliable, lightweight, small volume, with high efficiency, low electromagnetic interference and low current / voltage ripples^7,8^. In literature, in the power electronic field, there are two forms of interface converters: isolated and non-isolated interface converters^9^. In Ref^10^. , the hierarchical energy management strategy.

for FCHEV equipped with battery and SC was proposed. That includes two upper layers with the focus on SC power supply and lower layer to minimize fuel consumption.

In a vehicle energy system with a hybrid or a combination of multiple energy sources, managing the power flow is a very important indicator. The challenge is on the configuration and controller design that requires integration into existing systems within the vehicle to overcoming the complexity and difficulty resulting from. In this regard, the researchers have put their works on the agenda by considering the limitation of the charging status, assisting the FC during high loads, and optimizing the energy savings from the regenerative braking^11^. According to the growth of technology to apply FCs alongside battery / SC and photovoltaic in the automotive industry, but few articles have written to control these four sources together.

In Ref^12^. , an improved control strategy of active power distribution management between two FC sources and a SC bank has been proposed for the use of an EV with an electric motor structure inside the vehicle wheel (motor-wheel). The SC reference power is calculated by adjusting the DC bus voltage by the proportional integrator controller (PI). For FC required power, an algorithm is developed with five operating modes. This algorithm reduces the required power transient state depending on the SC state of charge (SOC) and vehicle speed level based on an aleatory driving cycle.

In Ref^13^. , power transmission elements have designed an FCHEV with the presence of FC/Battery/SC. The Energy Management Strategy (EMS) is achieved by providing a method for power-sharing. An intelligent control based on Fuzzy Logic Control (FLC) is implemented. Genetic Algorithm (GA) control parameters with respect to the goals and limitations of a multi-objective optimization performance on a city / highway driving cycle. It is based on Advanced Vehicle Simulator (ADVISOR) performed in EV-based research is one of the routine tasks. In Ref^14^. , a hybrid photovoltaic system has been introduced for EV’s battery charger, which introduces a simple maximum power point tracking (MPPT) for battery charging of EVs. In AC mode, an interleaved AC-DC boost converter with power factor correction (PFC) is also presented. This study utilizes a classical PI controller to regulate the voltage of DC-link using an interleaved converter used to convert alternating grid voltage to DC voltage at a common bus with PV.

Modeling, control and power management of hybrid Photovoltaic / FCs / Battery bank system supplying an EV is presented. While the PV and FC systems connected to the DC/DC converter as parallel. The battery bank is applied to store high energy as a floating-point connection without an electronic power converter. It examines the results of power allocation and power required estimation of a typical vehicle with direct torque control (DTC) strategy. But according to the important objectives of the hybrid-EV control system, in this work, it is not proceeded to independent control of the battery charge status as an important indicator. By relying on induction machine relationships, the results have not been evaluated for a specific driving cycle^15^.

In Ref^16^. , the study of a hybrid FC / battery system as an EV power has been done. The control system model for power allocation in this work is based on the measurement of the battery charge status and FC output power. It is investigated for a simple driving cycle with only two stops and two accelerations in the vehicle road and as an application for EVs. Experimental results are shown alongside the simulated Matlab / Simulink environment. In Ref^17^. , an energy management algorithm for a hybrid-EV with the low power scale of FC / battery / SC is presented. Based on a parallel coupling of sources to the DC common bus, a control system based on the measurement of the vehicle required current is obtained by the calculation of the power generated from the specific driving cycle. Also, measuring SC bank charging status as an energy source priority is determined to supply extra power at the moments of maximum power consumption from FC. It calculates the values of the converter reference’s currents to compare with their instantaneous values in the classical control system. Also, by selecting the battery bank as the vehicle’s energy supply at cruising speeds, it is caused the maintenance of the charge status by presenting results.

All of the above research pursues a specific structure and purpose. However, it is imperative to use a strategy that accomplishes all of the above goals in one structure. R1-1 & R2-1: The number of energy sources in most of the previous articles related to electric cars was 2 or 3. Considering that the characteristic curve of the sources is different and the optimal working point of each of the sources is different according to the working conditions of the car, the use of more and diverse sources to supply the energy needed by the car is a valuable suggestion. Therefore, in this article, the combination of fuel cell, battery, supercapacitor and solar panel sources is used to supply the energy needed by the car. In order to optimize the fuel, a new energy management system based on whale optimization algorithm (WOA) has been proposed to optimize the FOPI controller coefficients.

### The topological structure and characteristics of various FCHEVs

A summary and an analysis of the major characteristics about 4 types of FCHEVs is classified as shown in Table 1. Among these new FCHEVs based on PEMFC as main power source, the type of FC-Battery uses the battery as the peak power complement in the acceleration process, and absorbs the regenerative energy in the braking process. However, the voltage of FC does not adapt that of lithium battery, so it is usually connected by power converter. Another remarkable disadvantage of batteries is that their life span is extremely restricted^7^. In addition, When PEMFC is underpowered in FC-SC system, SC provides the energy required. SC can be charged and discharged at high speed, but it cannot be used for battery charging, so it cannot essentially regenerate the recycled energy. In addition, the price of SC is higher than that of battery, so the cost is a point to be considered in the system adopting SC^8^.

On the other hand, the system of FC-SC-Battery is more reliable than single power system because it concentrates the advantages of high energy density of battery and high-power density of SC. However, it costs more^13,18,19^. Also, there is a FC-PV-Battery that slightly is reliable rather than single power frame too, but it cannot be secure by low dynamic response. In despite of an area restriction on output surface of vehicle, it needs a high-gain converter to increase the small output voltage of PV source in connection with DC-Link^20^.

R1-1 & R1-2 & R2-1: This paper^21^ proposes a real-time cost-minimization energy management strategy for fuel cell/battery-based hybrid electric vehicles. It uses model predictive control, dynamic programming, and performance analysis to minimize operating costs and prolong fuel cell lifetime. The strategy reduces operating costs by 14.17% and 8.48% compared to a rule-based benchmark, and has a low online computation time per step. In^22^ compares two alternative tramway systems in Cuencae Ecuador, focusing on supercapacitors, lithium ion batteries, and proton exchange membrane fuel cells. The first uses renewable sources, while the second uses grid power. Energy and economic analyses are based on each system’s capacity and resources. This study^23^ integrated a hybrid hydrogen fuel cell system with a Li-Po battery into a UAV, determining power consumption and battery selection for both ground and air tests. In^24^, a solar-powered standalone charging station (CS) was constructed, using a 10 kW FC system as a substitute for two 6.5 kWh Li-ion battery banks. The CS’s theoretical concepts and MPPT technique were presented, and measurements were conducted under different weather conditions to verify its effective charge demand response. In order to guarantee the optimal operation of the recharging equipment used in electric vehicles, this study^25^ addresses an energy management issue. Finding the ideal conditions for managing a hybrid recharging system that combines fuel cells with solar cells is the major goal of this work. Two converters connected the fuel cell and photovoltaic systems in parallel so as to supply power to the main traction motor or a bank of lithium batteries. This study^26^ compares the energy distribution of fuel cell electric vehicles versus fuel cell hybrid electric vehicles. Fuel cell electric vehicle hybridization is developed with a 15 kW traction battery installed. In order to facilitate regular determination, modeled automobiles with identical fuel cell stacks and similar chassis were built under the AVL Cruise initiative. A comparison task was used to show and graph numerical analyses along with instantaneous findings in terms of Sankey diagrams.

**Table 1:** Summary and analysis of the major characteristics about 4 types of FCHEVs.

FC-HEVs	Power Density	Energy Density	Dynamic Response	Charging Time	Driving Range	Ref.
FC-Battery	Low	High	Low	Low	Short	^7^
FC-SC	High	Low	Medium	Medium	Short	^8^
FC-SC-Battery	High	High	High	Fast	Long	^13,18,19^
FC-PV-Battery	Low	High	Low	Low	Medium	^20^

R1-1 & R1-2 & R2-1: This paper presents a significant advancement by introducing a new energy management system that integrates fuel cells, photovoltaic panels, batteries, and supercapacitors. The use of a high-step-up DC/DC converter and MPPT algorithm for adapting the power output from solar panels ensures optimized performance and enhanced system efficiency. Additionally, the implementation of an FOPI controller and evolutionary optimization algorithms for more effective energy management, along with validated simulation results under real-world driving conditions, demonstrates the high performance and capability of your system to deliver optimal performance in practical scenarios. Table 2 shows the analysis of a number of references.

**Table 2:** Analyzing a number of references.

References	Number of energy source	Method	Advantages	Disadvantages
^21^	3	Model Predictive Control	Real-time optimization	Implementation complexity
^22^	2	PEMFC	Reducing emissions	High initial costs
^23^	2	Natural Gradient	Reducing the weight of the energy system	Complexity of design and maintenance
^24^	2	Natural Gradient	Fuel cell support in critical times	Dependence on weather conditions
^25^	3	Adaptive Network-based Fuzzy Inference System	High efficiency	High costs
^26^	2	Adaptive Network-based Fuzzy Inference System	Improved stability and performance	System complexity

According to the status of the sources framework as mentioned in Table 1, it’s considered four resources of FC, PV, SC, and Battery in this paper to supply more advantages. However, they have different features with instantaneous variations of vehicle’s energy requirements that makes to utilize a suitable management algorithm as an essential challenge. To solve this problem, we have tried to propose an integrated management algorithm that helps to the sources to being at their optimal conditions and fuel saving. Also, a standard urban driving cycle is considered to respond appropriately to the vehicle’s power demand. The configuration of the vehicle power supply is provided in parallel with the power sources. They connected by power converters to the common bus which is utilized by a high-efficiency DC/DC converter due to the low power of the PV. The energy management algorithm generates reference currents for the converters by a FOPI controller. We consider the limitation of battery current to distribute energy between storage sources. In this work, the Whale Optimization Algorithm (WOA) is used to find the optimal control parameters derived from the energy management algorithm. The energy management algorithm is shown to confirm the work. Finally, the results of the simulation are validated in Matlab / Simulink.

**Fig. 1:**
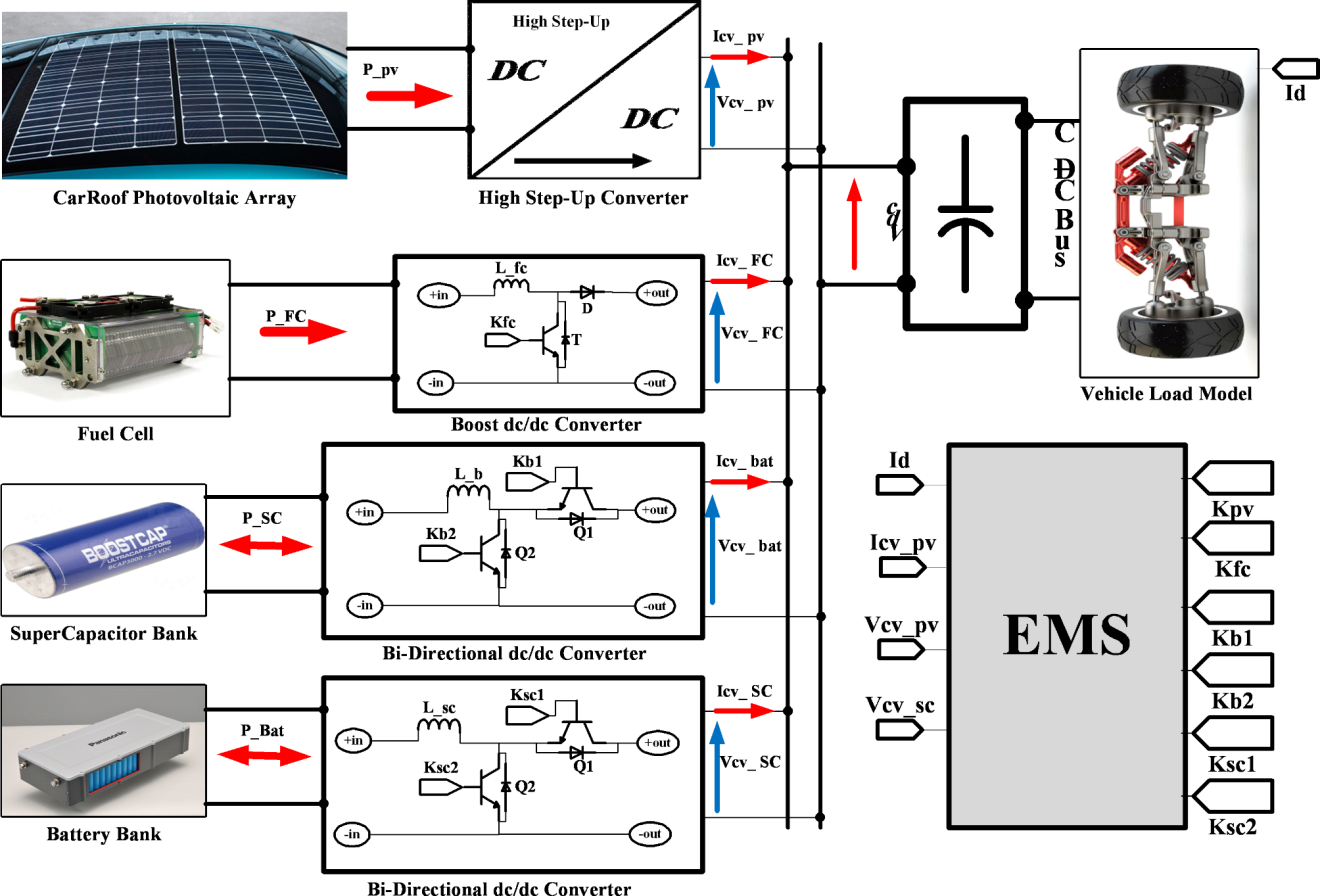
Typical electric vehicle system.

## Configuration of studied FCHEV

The studied EV system consists of four sources by FC, photovoltaic, and battery / SC, which are responsible for supplying the energy needed to drive the vehicle in various driving cycles. Figure 1 shows an overview of the vehicle’s electrical system under study. In the output of the photovoltaic source, a high step-voltage DC/DC converter is used to convert the low-voltage photovoltaic source to an optimal value in the common bus. The unidirectional DC/DC converter (Boost) is used for the FC source to boost the voltage level to the expected value and keep it constant at the output value. A bidirectional DC/DC converter (Buck / Boost) uses a variable amount of voltage in a battery bank such as SC bank to increase the constant voltage to assist them in a bidirectional state of energy transfer.

### Power of electric machine and fuel-cell source

In these systems, at cruising speeds and without the use of secondary sources, the FC source power is approximately 65% of the maximum power required by the electric motor in the vehicle. Due to the limitations of the vehicle acceleration, speed, slope angle, and the aerodynamic or dynamic effects of the Eq. (1), the vehicle’s load can be expressed as follows^27^:

1 $$\:{P}_{Load}=\frac{V}{{\eta\:}_{t}}\left({M}_{v}{gf}_{r}cos\alpha\:+\frac{1}{2}{\rho\:}_{a}{C}_{D}{A}_{f}{V}^{2}+{M}_{v}{gf}_{r}sin\alpha\:+{M}_{v}\delta\:\frac{dV}{dt}\right)+{P}_{aux}$$

Where $\:\delta\:$ and $\:{\eta\:}_{t}$are the coefficient of the mass of the rotating elements and the power transfer efficiency of the vehicle, respectively. And $\:{P}_{aux}$ is auxiliary power for automobile auxiliary uses such as lighting, beep, etc., which are usually around 200 to 300 watts. The basis for calculating the electric motor power in a passenger car is maximum cruising speed and vehicle acceleration. Table 1 illustrates the typical vehicle used in this work. To calculate the acceleration time (usually 0 to 100 km/h in a passenger car), we must obtain the Eq. (1) for time t. Given the number of variables of the Time-Acceleration equation and their dependence on the speed of the vehicle, the curve $\:({t}_{a}-{P}_{m})$ can be shown only using numerical calculations for a typical vehicle with the specifications of Table 3, as shown in Table 4. According to Eq. (1) and Table 5, to provide the vehicle required power for acceleration within 12 s, the maximum required power by an electric motor is about 70 kW. In a FC hybrid-EV power system, the primary or main power is provided by the FC, which determines the power ($\:{P}_{e}$) of the vehicle at cruising speed or driving on a road with a specified class based on Eq. (2):

2 $$\:{P}_{e}=\frac{V}{{\eta\:}_{t}{\eta\:}_{em}}\left[{M}_{v}g{f}_{r}{cos}(\alpha\:)+0.5{\rho\:}_{a}{C}_{D}A{v}^{2}+{M}_{v}gi\right]$$

Where i is road grade or the ability of the vehicle to move from slopes and V is the speed of the vehicle based on the real driving cycle. The FC must be able to provide equal energy for the vehicle in the state of the cruising speed.

**Table 3:** Typical vehicle characteristics.

Characteristic	Parameter	Unit	Value
Vehicle Mass	$\:{M}_{V}$	$\:kg$	1500
Mass coefficient of rotary elements	$\:\delta\:$	–	1.043
drag coefficient	$\:{C}_{D}$	–	0.335
Rolling resistance coefficient	$\:{f}_{r}$	–	0.015
Gravity	g	$\:\frac{m}{{s}^{2}}$	9.81
Air density	$\:{\rho\:}_{a}$	$\:\frac{kg}{{m}^{3}}$	1.202
Road angle	$\:\alpha\:$	Degree	2
Vehicle front area	$\:{A}_{f}$	$\:{m}^{2}$	2
Efficiency of electric mtor	$\:{\eta\:}_{em}$	–	0.92
Efficiency of transmission system	$\:{\eta\:}_{t}$	–	0.9
Efficiency of machine	$\:{\eta\:}_{m}$	–	0.85

**Table 4:** Details of numerical calculation to obtain power requirement electric motor ($\:{P}_{m}$) based on acceleration time ($\:{t}_{a}$) for a typical vehicle^1^.

$\:{t}_{a}$ (sec)	52.5	45	32.5	22.5	17	15	13	12	10	8
$\:{P}_{m}$ (kW)	22.5	25	30	40	50	55	60	70	80	100

Therefore, according to the information of Table 4 and Eq. (2), the maximum suitable power for the FC source would be 50 kW based on typical vehicle characteristics which is used the type of proton exchange membrane FC (PEMFC) that is a suitable choice for vehicular applications based on proper operating point of power and temperature^1^. The typical FC source specifications are shown on Table 6.

**Table 5:** Guide to calculating the FC source power.

Vehicle Speed		Road angle $\:\varvec{\alpha\:}$ (Deg)	Road grade i	Vehicle electric power $\:{\varvec{P}}_{\varvec{e}}$،(kW)
Speed Type	mile/h			
Normal	60	0	0%	13.1
Max	95	0	0%	42.1
Cruising	55	0	10%	50

**Table 6:** Typical PEMFC power source characteristics.

Character	Value	Character	Value
Power rated	35.78 kW	Oxygen pressure	1 Bar
Max power	50 kW	Hydrogen pressure	1.5 Bar
Rated voltage	270 V	Temperature	℃ 65
Resistance	Ω 0.47	Cell number	390

### Photovoltaic source

Photovoltaic source modeling is proposed in the typical vehicle system assuming at least $\:1{m}^{2}$ of roof area. Japanese company Panasonic has developed the full solar roof for the Japanese version of the Toyota Prius Prime plug-in hybrid. Panasonic claims that this new version can produce more power than before, making it easier to mount on a curved roof^28^. The power of this source is considered as auxiliary power ($\:{P}_{aux}$) of the vehicle. It have effects on improving the overall response of the vehicle required power as well as improving fuel consumption. Table 7 presents the solar panel specifications based on vehicular usage.

**Table 7:** Solar panel specifications of Sun perfect solar CRM200S156P-54.

Character	Parameter	Value
Max power (w)	$\:{P}_{max}$	200
Short circuit rated current (A)	$\:{I}_{sc}$	8.21
Open circuit rated voltage (V)	$\:{V}_{OC}$	32.9
MPP current (A)	$\:{I}_{max}$	7.5
MPP Voltage (V)	$\:{V}_{max}$	26.6
Voltage Temperature Coefficient (%)	$\:{K}_{V}$	12.3-
Current Temperature Coefficient (%)	$\:{K}_{i}$	0.32
Cell number	$\:{N}_{S}$	54
Resistance(series-parallel) (Ω)	$\:{R}_{p}-{R}_{S}$	0.221- 415.405

### Secondary power source

Li-ion batteries have a better response than lead-acid, nickel-cadmium or nickel-metal hybrids. Also, they are lighter, more energy-saving, and have a higher life cycle and reliability^18^. According to the maximum power of the selected electric motor based on the specific acceleration performance as well as the powers of FC and PV are based on driving conditions at a constant speed, the sizing of the secondary power source can be expressed as follows:

3 $$\:{P}_{\text{Secondary}}=\frac{{P}_{m}}{{\eta\:}_{m}}-\left({P}_{FC}+{P}_{PV}\right)\:\:\:\:\:\:\:\:$$

Given the above Equation, the battery storage power will be around 33 kW. Depending on the characteristics of each battery cell, the cells number of battery can be obtained from the following relation:

4 $$\:{N}_{b\_cell}=\frac{{P}_{{sec}ondary}}{{m}_{cell}\times\:{P}_{specific}}\:\:\:\:\:\:\:$$

According to Table 8, at least 28 battery cells will be needed to build the battery bank assembly to support the vehicle required power at the start of the movement (given the time of FC warming to generate power).

### Supercapacitor

Based on the dynamic characteristic of the FCHEV power sources, the FC power generation capability is limited to initial warming up. Therefore, at the start or acceleration of the vehicle’s movement, the storage system is responsible for propulsion the vehicle with a fast power transmission. Battery power at the middle dynamics rate will have a maximum discharge, assuming that itself supplies the vehicle with the necessary acceleration power. Therefore, the supercapacitor bank with the high power density alongside the battery can be the perfect combination to meet the vehicle’s need at the acceleration and start of a movement. Based on the maximum discharge energy required to supply 33 kW in 12-second acceleration time, approximately 110 Wh energy is required. The total energy capacity of the SC bank can be expressed as (5):

5 $$\:{C}_{E}=\frac{\varDelta\:{E}_{max}}{{C}_{p}}\:\:\:\:\:\:\:\:\:$$

Where $\:{C}_{p}$is the percentage of total energy capacity that is allowed to be used. When using the SC bank, if the maximum voltage drop at the terminal be 20%, $\:{C}_{p}$is considered to be approximately 0.36. With this description and attention of Table 9, 152 SC cells are needed for use in the automotive system.

**Table 8:** Li-ion battery bank specifications.

Character	Value
Rated voltage	3.3 V
Rated capacity	19.5 Ah
Specific energy density	131 Wh/kg
Specific power density	2400 W/kg
Cell weight	0.496 Kg
Cell number	28

**Table 9:** SC bank specifications.

Character	Value
Rated capacity	2000 F
Max capacity (primary)	2400 F
Max series resistance	0.35$\:m\varOmega\:$
Rated voltage	2.7 V
Max absolute voltage	2.85 V
Specific energy density	5.6 Wh/kg
Series cell number	152
Cell weight	0.36 Kg

### High step-up DC/DC converter

**Fig. 2:**
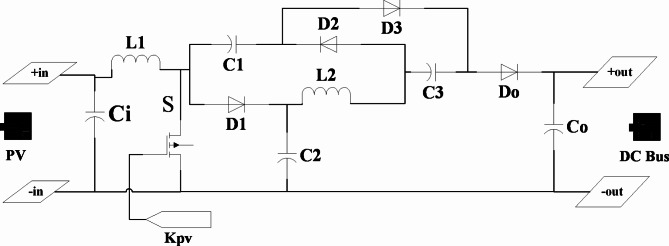
The Proposed schematic circuit of single switch unidirectional high-step up converter.

Due to the output low voltage range of the solar panel that is used in the roof of the car, high-step up converter must use. Single-switch boost converters, depending on their structure, can solve the problem of induction large leakage current at the output of converters that are created if transformers will be used. Also, without the requirement of filter design, it will not make problems in high frequency switching. But this condition affects the resonance of the switches voltage and increases the weight, volume and size of the converter^29^. Depending on their feature, single-switch boost converters can produce output voltages equal to 30 times the input voltage, and given the low voltage range of the photovoltaic panel used at the car roof (around 30 volts) can be a good choice for the proposed model in the configuration of the EV power system that is outlined in this paper. Figure 2 shows the circuit schematic of the converter. The converter’s specifications are shown in Table 10. The input-output relation of the converter is as Eq. (6):

6 $$\:\frac{{V}_{DC}}{{V}_{pv}}=\frac{3+D}{2(1-D)}\:\:\:\:\:$$

**Table 10:** Proposed single switch unidirectional high-step up converter specifications to photovoltaic source.

Character	Symbol	Value
Max output power	P	250 W
Input voltage	Vpv_max	27 V
Output voltage	Vout	390 V
Switching frequency	Fs	24 kHz
Inductance	L1 and L2	205 uH and 180 uH
Capacitor	C1, C2, and C3	2.08 uF
Output capacitor	Co	140 uF

## Comprehensive energy management system

The control strategy must ensure that the delivered power of the electric motor at all times provides the required power. For be simplicity, the demand response of the electricity system is accepted instantaneously. The control strategy is to obtain the required power by FC ($\:{P}_{FC}$), photovoltaic ($\:{P}_{pv}$), battery bank ($\:{P}_{Bat}$) and SC ($\:{P}_{SC}$) to provide the demand of the vehicle without knowing the future driving conditions. Therefore, the following equation can be expressed:

7 $$\:{P}_{d}={P}_{FC}+{P}_{pv}+{P}_{Bat}+{P}_{SC}\:\:\:\:\:\:\:\:\:\:$$

### Proposed energy management algorithm of power sources

In this paper, while we proposed an energy management algorithm (EMA) that is a main function for the energy distribution between the four power sources while the presence of the photovoltaic source is influenced by factors such as being night / day and limiting the battery charge status. Therefore, the selection of sources that provide the required power with 4 switches ($\:{K}_{CV\_FC\:},\:{K}_{CV\_PV},\:{K}_{CV\_Bat},\:and\:{K}_{CV\_SC}$) is permitted for FC, PV, battery and SC respectively. The control of the energy flow between the main sources of FC and the PV with the storage system is dependent on the required current ($\:{I}_{d}$) and the supercapacitor voltage. The rule on which we based our work, is the limitation of the battery current. So the SC provides the difference between the required current by the vehicle and the sum of the current supplied by the battery, which is limited to an imposed value, and the current given by the FC and PV (Eq. (8)).

8 $$\:{I}_{CV\_SC}={I}_{d}-({I}_{C{V}_{PV}}+{I}_{C{V}_{FC}}+{I}_{CV\_Bat\_max})\:\:\:\:\:$$

**Fig. 3:**
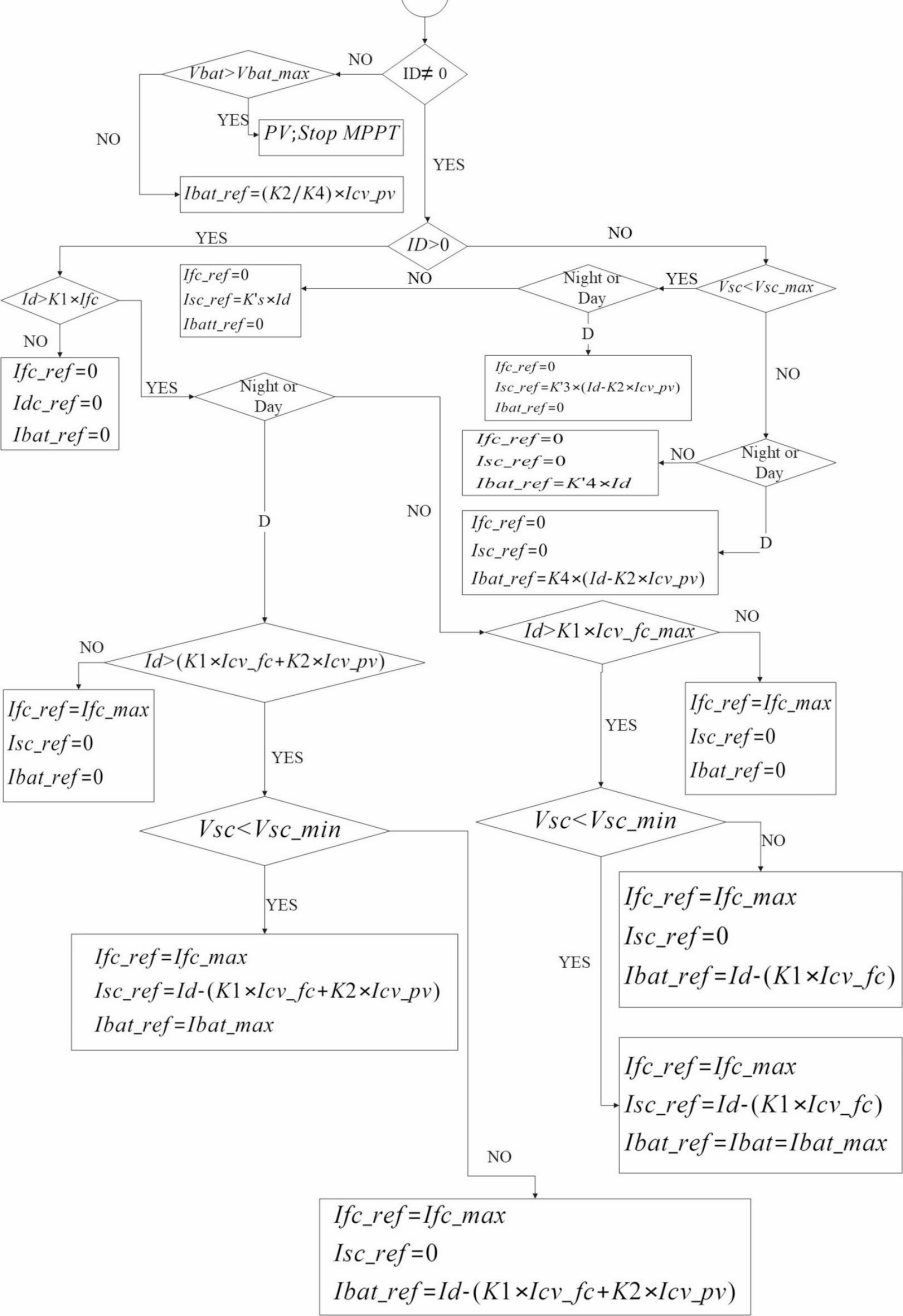
Energy management algorithm of power sources.

The EMA is shown in Fig. 3 Since the SC can be easily charged and discharged compared to the battery; in this study we used the battery source only in high required power conditions. According to the algorithm of Fig. 3, as long as the required power ($\:{P}_{d}$) is less than the sum of the FC and photovoltaic power (unless there is a night limit or battery SOC), the FC and PV act as the only energy sources; As the required power is greater than the sum of the power of these two sources, the FC operate at its rated power and the photovoltaic at their maximum power, and the rest is supplied by the SC. If the required power exceeds the sum of the FC, PV and SC power, the battery is also used. Note that the battery and SC are limited to operating within their predefined range of charge status (SOC). While the vehicle moves and required power is lower than the sum of the nominal FC and PV power, the two secondary storage systems can be loaded using additional FC energy. In braking mode, charging the battery or SC depends on the degree of deceleration. At the weak negative acceleration, the situation is exploited to charge the battery, while at strong negative acceleration due to higher power density, the charging is used to charge the SC. In this study, according to Fig. 1, the vehicle load model is considered. In this section, the load model is considered as a dependent current source whose reference of the vehicle required current ($\:{I}_{d}$) constitutes the signal of that to determine the characteristics of the required power variations of the vehicle for the power allocation system. And with the required power by the vehicle, the power set of energy sources can provide the vehicle’s load demand. According to Table 10, which shows the coefficients related to the power supply converter current with the DC common bus side, the algorithm presented in Fig. 3 is classified according to the following states:

1. *Determine the time of day or night*: This condition indicates the available (day time) / unavailable (night time) of the photovoltaic system to generate optimum power. In the simulation, this is done by considering a two-state relay (+ 1/-1) with a threshold whose first input is the output duty cycle pulse of the P&O system from the maximum power point algorithm. The second input of that is the signal of maximum duty cycle pulse for short-circuit of switch by 24 kHz frequency (Fig. 2).
2. *Vehicle Park Mode*: This occurs when the vehicle has been stopped for a long time and obviously the amount of required current by the vehicle ($\:{I}_{d}$) will be zero.
3. *Positive acceleration mode*: These include (a) Overload mode, (b) SC bank charging status, (c) Battery charging status, and (d) Full vehicle stop mode (in very short time).
4. *Negative acceleration mode*: Includes charging status of SC Bank / Battery Bank.

However, the maintenance strategy of the charge status is usually chosen to prevent the battery from depleting completely at the end of the period. As such, the user does not need to charge the system like an EV because the battery state always remains above the minimum charge level. To achieve this, the final battery charge status is monitored and compared to the initial value. The EMA presented in Fig. 3 starts by measuring the required current ($\:{I}_{d}$) and calculates the reference current values for the FC, battery and SC, respectively, $\:{I}_{FC\_ref}$, $\:{I}_{Bat\_ref}$and $\:{I}_{SC\_ref}$at the outputs, based on a standard driving cycle.

### FOPI controller and proposed optimization algorithm

To achieve suitable power transmission and operation of any power source, the difference between the power converters output currents and their reference values shall be minimized; however, reference values obtained from the EMA. Table 11 shows Coefficients of Conversion of Current Components of Power Converters Applied to Energy Management Algorithm. For this purpose, in this section, we use three fractional-order proportional-integral controllers (FOPIs) for FC, SC, and battery bank respectively to minimize the difference between the reference and operating (real) values of the currents by high accurate. And the controller output using pulse width modulation (PWM) converted to pulse signals for the switches of the power converters.

**Table 11:** Coefficients of Conversion of Current Components of Power Converters Applied to Energy Management Algorithm.

Converter		symbol	value	Example
FC		K1	0.56	Id = K1*Ifc
PV		K2	0.077	Id = K2*Ipv
SC	charge	K3	0.2	Id = K3*Isc
	discharge	Kˊ3	5	Isc = Kˊ3*Id
Battery	charge	K4	0.25	Id = K4*Ibat
	discharge	Kˊ4	4	Ibat = Kˊ4*Id

The whale optimization algorithm (WOA) has been used to optimize the FOPI controller coefficients. In this algorithm, while producing the optimal coefficients of the FOPI controller, it is also designed in a suitable control loop for the DC common bus output voltage to follow the reference value of 400 volts. Since the first-order FOPI has a more parameter than a conventional PI controller, more characteristics can be provided. It improves system performance and increases robustness against energy source uncertainties, such as increases and variations of the time constant^19^.

The FOPI controller has more flexibility with high precision matching in closed-loop control systems. The FOPI transfer function is obtained by Laplace transform and is as follows:

9 $$\:G\left(s\right)={k}_{p}+{k}_{I}{s}^{-\lambda\:}\:\:\:\:\:\:\:\:\:\:\:\:\:\:\:\:\:\:\:\:\:$$

The FOPI design is consisted of specifying two indices $\:{k}_{p}$and $\:{k}_{I}$, and a rank $\:\lambda\:$ which is not necessarily an integer^30^. The behavior of the WOA algorithm can be illustrated by the mathematical model as follows:

10 $$\:\overrightarrow{D}=\left|\overrightarrow{C}.\overrightarrow{{X}^{*}}\left(t\right)-\overrightarrow{X}\left(t\right)\right|\:\:\:\:\:\:\:\:\:\:\:\:$$

11 $$\:\overrightarrow{X}\left(t+1\right)=\overrightarrow{{X}^{*}}\left(t\right)-\overrightarrow{A}.\overrightarrow{D}\:\:\:\:\:\:\:\:\:\:\:\:\:$$

where t indicates the current iteration, $\:\overrightarrow{A}$and $\:\overrightarrow{C}$are coefficient vectors, $\:{X}^{*}$ is the position vector of the best solution obtained sofar, *X* is the position vector and $\:\overrightarrow{D}$is the distance vector between the best position vector and the previous iteration position vector. It is important to note that according to Eq. (11), the $\:{X}^{*}$must be updated in each iteration to find the best final position^30^. In the proposed WOA algorithm to solve the optimization problem, the following function is used as the basic function of classical and representation functions:

12 $$\:F\left(x\right)={\sum\:}_{i=1}^{n}\left|{x}_{i}\right|+{\prod\:}_{i=1}^{n}\left|{x}_{i}\right|\:\:\:\:\:\:\:\:\:\:$$

Where *n* is population of whales^31^. The values of the FOPI control indices and other information about the WOA algorithm are presented in Table 12.

**Table 12:** Parameters of FOPI Controller and WOA Algorithm.

FOPI Controller			
Parameter	FC	Battery	SC
$\:{k}_{p}$	0.8151	0.9957	0.3428
$\:{k}_{I}$	0.7032	0.5812	0.999
$\:\lambda\:$	1.124	1.8074	0.565
WOA Algorithm			
Variation Range	[-0.999, 0.999]	Iteration	15
Frequency Range	[0.001, 1000]	Population	25

### MPPT algorithm of PV source

**Fig. 4:**
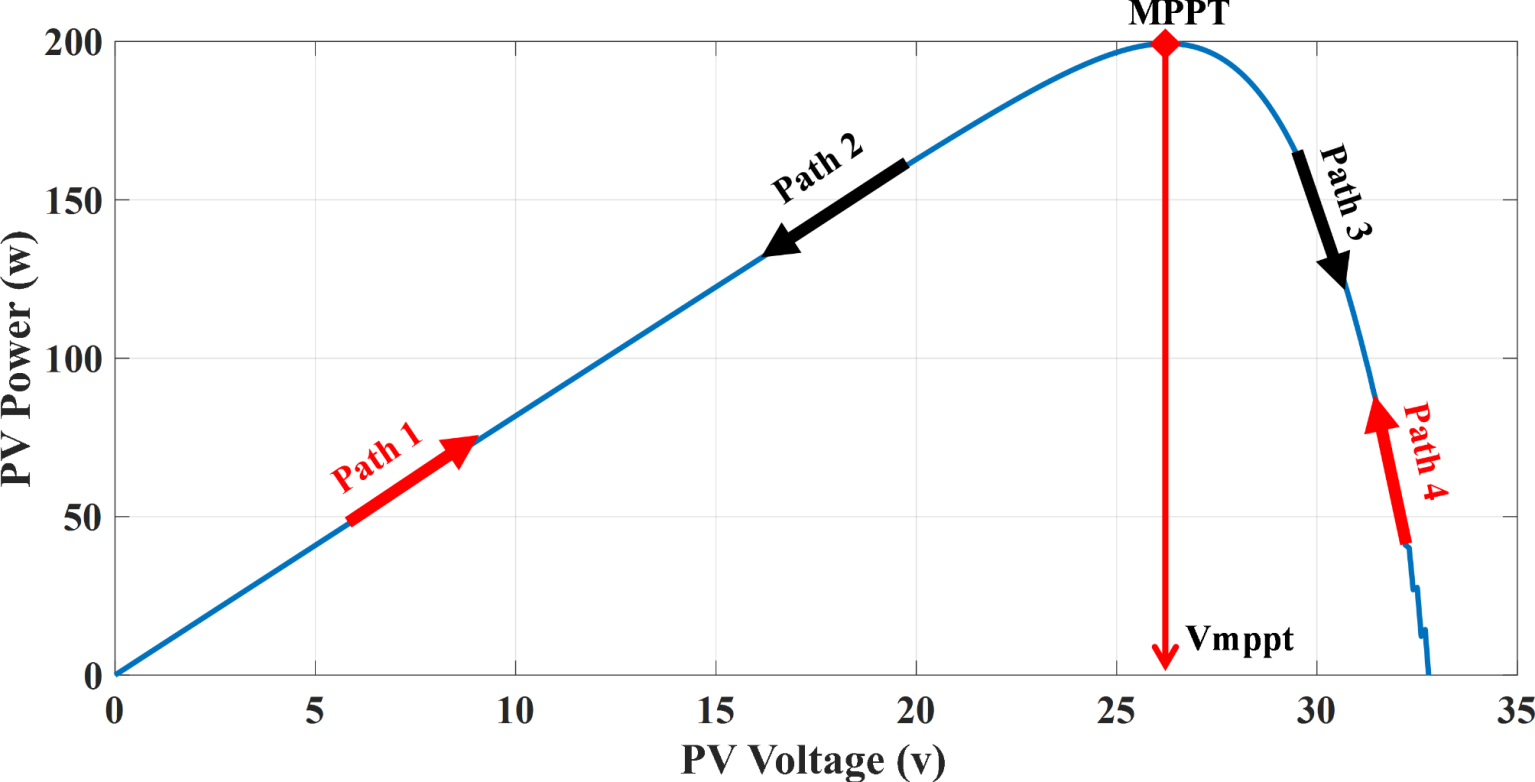
Routing technique for photovoltaic source MPPT algorithm^32^.

In this section, based on the observation and perturbation (P&O) algorithm, due to assuming no partial shadow problem, at first by calculating the values of the voltage and current of the solar panel, it first calculates the power value at any given moment (P(k)) then by comparing it to a moment ago (P(k-1)) in programmed circles it goes on. In this method, the power value of PV will be maximum if the value of power’s subtraction be equal to zero at these two moments. On the other hand, by checking the sign of the power subtraction’s value, it must be calculated for both positive or negative sign, the subtraction’s value of PV voltage (V(k)) with a moment ago (V(k-1)). By inspecting this, four paths will be determined from the P-V curve (Fig. 4):

1. If $\:P\left(k\right)-P(k-1)>0\&V\left(k\right)-V(k-1)>0\Rightarrow\:$ The reference of PV voltage must be increase. (Path 1)
2. If $\:P\left(k\right)-P(k-1)>0\&V\left(k\right)-V(k-1)<0\Rightarrow\:$ The reference of PV voltage must be decrease. (Path 4)
3. If $\:P\left(k\right)-P(k-1)<0\&V\left(k\right)-V(k-1)<0\Rightarrow\:$ The reference of PV voltage must be increase. (Path 2)
4. If $\:P\left(k\right)-P(k-1)<0\&V\left(k\right)-V(k-1)>0\Rightarrow\:$ The reference of PV voltage must be decrease. (Path 3)

Thus, according to the P-V curve of the PV source to be at the maximum point, the four paths must go through which the two paths are incorrect and must be corrected and the other two paths are correct as shown on Fig. 4 clearly. Now according to Eq. (6), the duty cycle value of the photovoltaic converter has a direct relation with the PV source voltage that is converter’s input and so the process will continue. Figure 4 illustrates the routing technique for the MPPT algorithm of the photovoltaic source in four paths, which are paths 1 and 4 correct, and paths 2 and 3 are the wrong paths for tracking the maximum point^32^.

## Simulation results

**Fig. 5:**
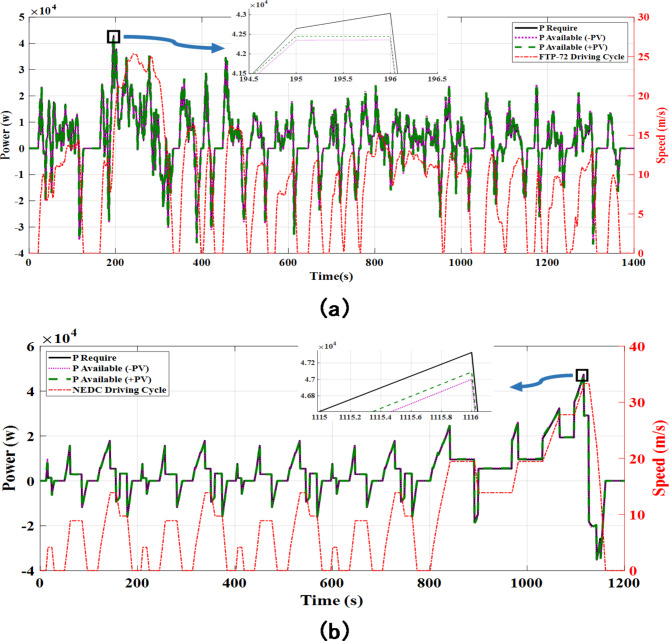
Displaying driving cycles and required power curve with the influence of PV power for the vehicle power required response together for FTP-72 (**a**) and NEDC (**b**).

In this section a detailed operating model is presented which is presented in Tables 1, 4, 5, 6 and 7 together with the dynamic model of vehicle load. In order to prove the studies in this paper, some simulations have been carried out. To control the process of the energy management system in the proposed vehicle model, we have selected a standard urban driving cycle of Federal European Test Procedure (FTP-72) that has a high number of stops and accelerations (Fig. 5(a))^33^. Based on Eq. (1), the required power of the vehicle during the proposed driving cycle will be as shown in Fig. 5(a) too. It also is shown to New European Driving Cycle (NEDC) in Fig. 5(b) to valid performance of proposed energy management system.

The scenario used in this work is due to the role of the PV source in the overall estimation of the vehicle power required response. And in other hand, from a specific perspective, as mentioned to the term auxiliary power ($\:{P}_{aux}$) in Eq. (1), the PV source can be used to estimate this term from the total demand for vehicle power. For this purpose, given the limitations such as night time or weather conditions, in practice to use of the PV source should use additional equipment such as a light sensor in the vehicle framework but in design and simulation to show the appropriate output, whilst the time period of FTP-72 driving cycle is around 23 min, we validate our work by the available / unavailable of a PV source.

**Fig. 6:**
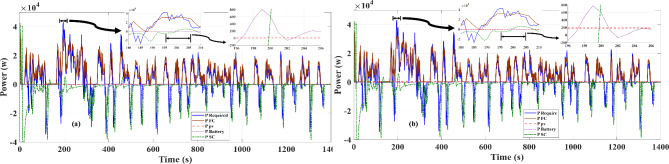
Dynamic response of power resources to required power with participation influence of PV source in vehicle’s set (**a**,**b**) in FTP-72 Driving Cycle.

For this purpose, it can be seen to improve the overall response to the vehicle required power of about 110 watts from Fig. 5 that is a comparison to showing the influence of PV power for the vehicle power required response. To investigate the dynamic manner of the FC, PV source, battery, and SC in response to the total required power ($\:{P}_{d}$), Fig. 6 is presented for the available (a) or unavailable (b) of PV source, respectively. As shown in this figure, due to the high-power scale of a battery bank, but in response to the vehicle’s power set, the battery has a lower share than other sources; in the EMA, the power response priority between the capacitor and battery sources is taken into account for the capacitor bank. And on the other hand, the battery bank, according to the proposed algorithm policy (Eq. (8)), due to its high energy density as a stabilizer of the vehicle’s demand energy is intended at acceleration points (time range of 190 to 240 s in the FTP-72 driving cycle as the largest acceleration ).

**Fig. 7:**
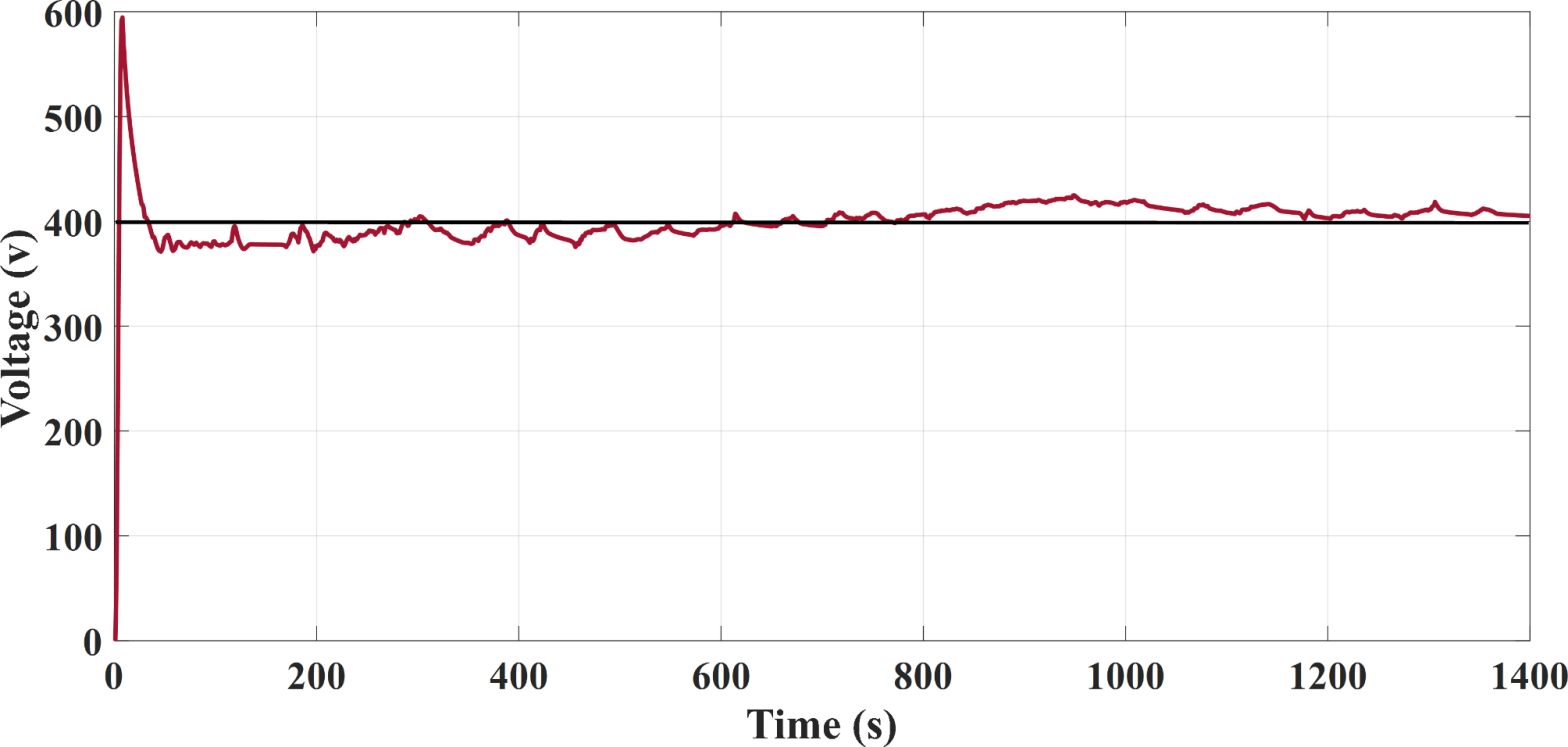
DC-bus common voltage (FTP-72).

**Fig. 8:**
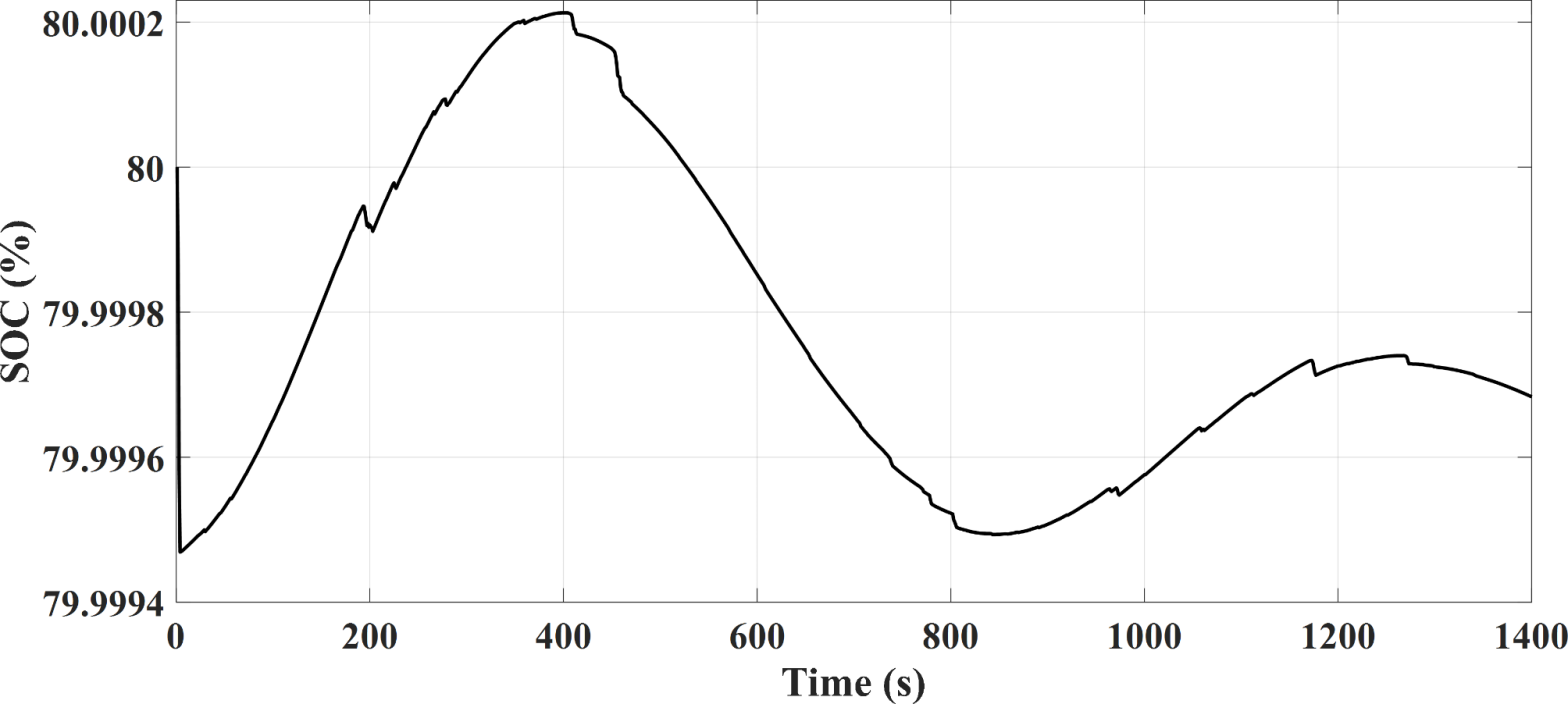
Battery Charging Status in FTP-72 Driving Cycle.

**Fig. 9:**
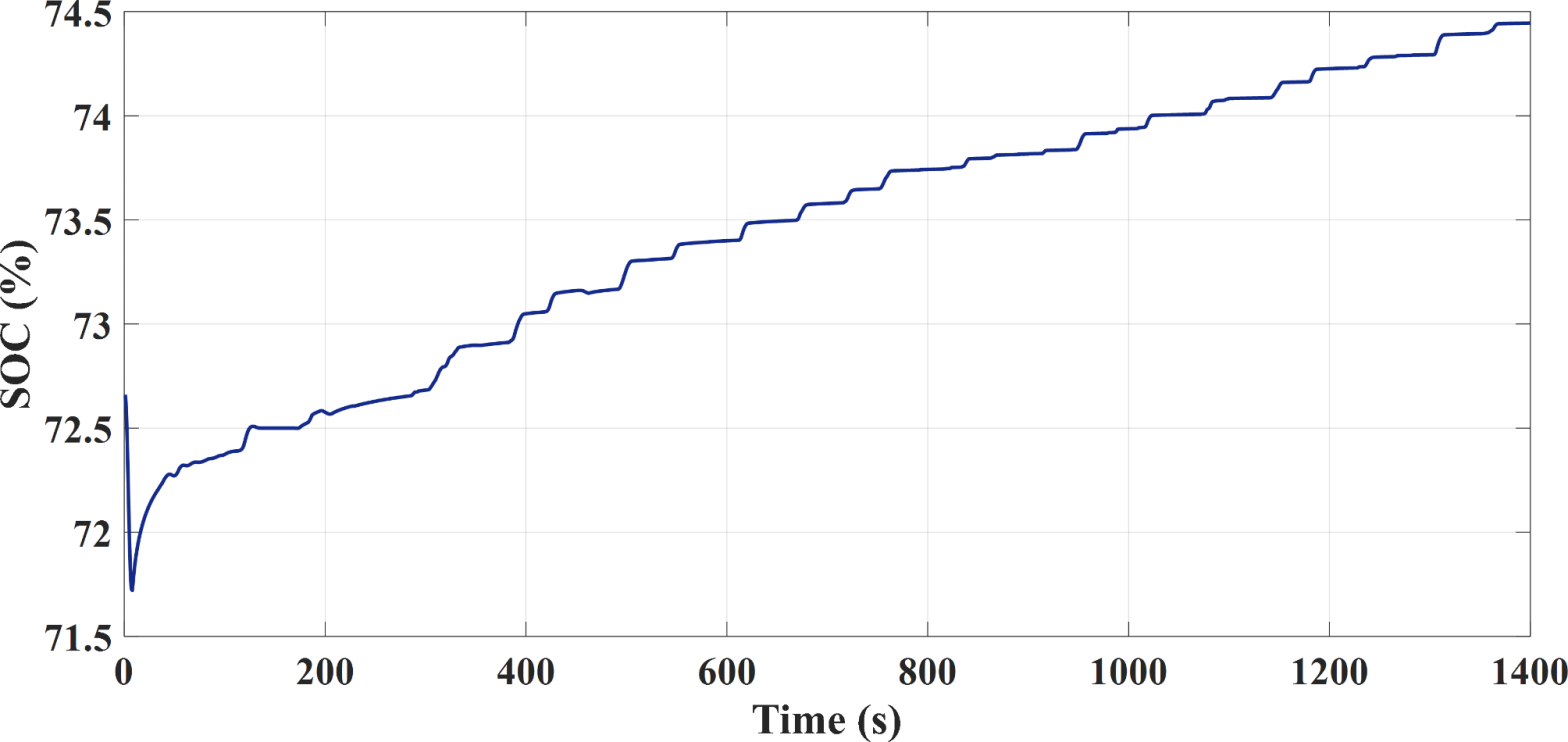
Super Capacitor Charging Status in FTP 72 Driving Cycle.

The required power is located on a higher level at the acceleration time. The FC source generates maximum power at operating point and the PV source generates maximum power in the unavailable state of mentioned limitations, and the secondary storage system generates the lack of the required energy to the vehicle in this case to assist the main sources of vehicle power. The generation (discharges) or not-generation (recharges) from the battery or SC is by the two switches K1 and K2, respectively as shown in Fig. 1. According to the EMA and the optimization algorithm, the results shown from Figs. 7, 8 and 9 are almost similar in both available / unavailable condition of a PV source. Battery charging range has settled from 65 to 80% and also for SC range from 40 to 90%. As shown in Figs. 8 and 9, respectively, the SOC of the energy storage system is maintained.

According to Eq. (8), there are two attention from limiting the battery current and supplying the difference between the required current and the sum of the resources currents by the SC bank. Also, that is considered the SC voltage in algorithm of Fig. 3 as the charge / discharge priority at acceleration / braking points than the battery bank, due to the positive / negative trend from charging status for acceleration / braking points during the driving cycle, respectively. Based on above matters, there is a 2% increase in charge compared to steady state. Also in Fig. 6, according to the proposed EMA, the SC bank in acceleration cycles despite the limitation of the battery current, provides the amount of vehicle required power in negative acceleration that the use of the regenerative braking energy is the result of that.

## Study of saved fuel

**Fig. 10:**
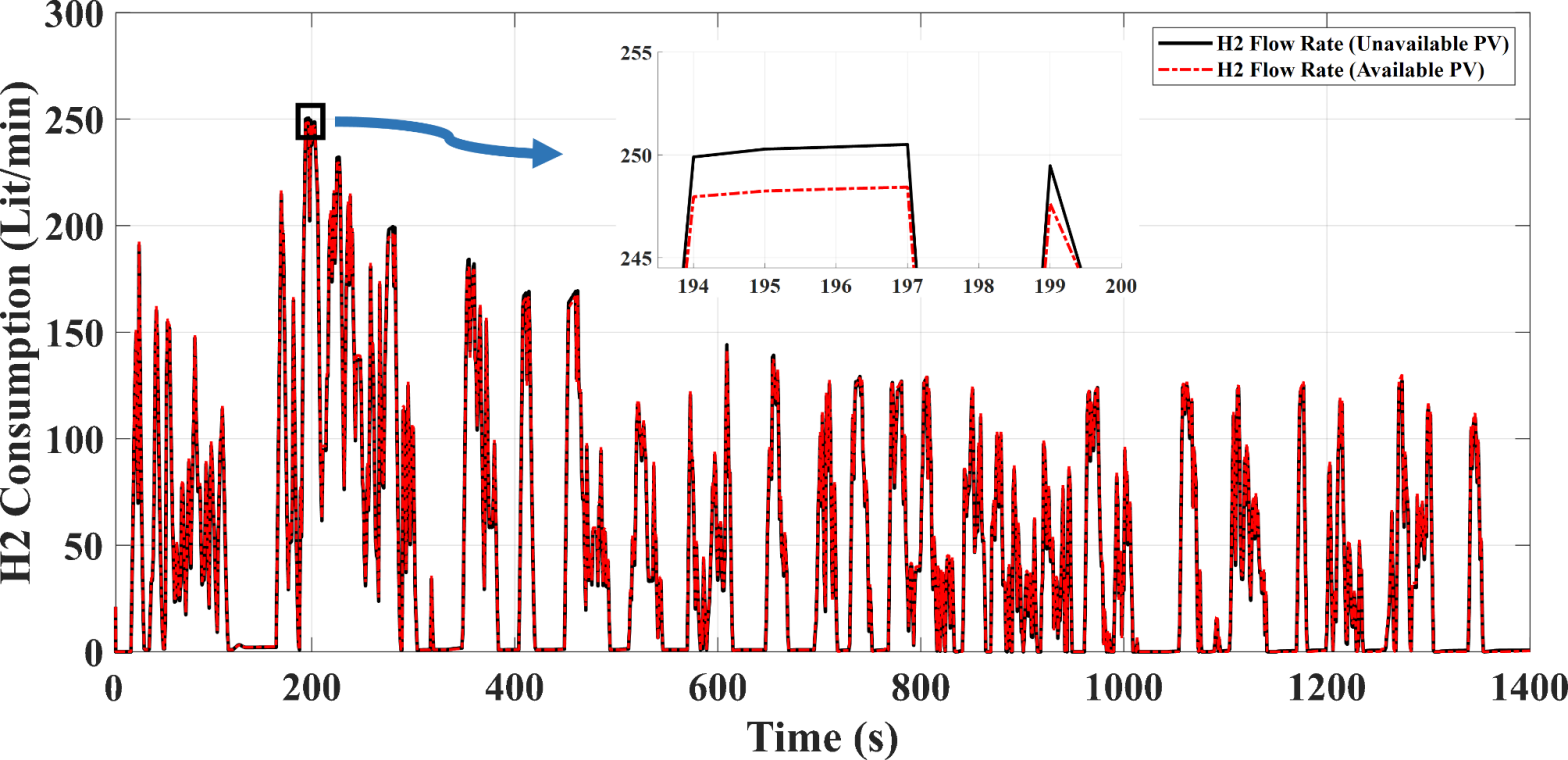
Display of hydrogen fuel consumption curve by FC source in the presence of photovoltaic source based on FTP-72 driving cycle.

According to the fact that the calculations of this part are based on the effects of using the free source of solar energy installed on the roof of the car, the performance of the saved fuel is supposed to during one month that according to the obtained output from the hydrogen consumption curve of vehicle that is relied on Eq. (13) as shown in Fig. 10 that will be followed by:

- The maximum time of sunlight is equal to the time period of the selected driving cycle per day;
- The calculations of fuel consumption performance are determined as monthly.

The hydrogen consumption rate of fuel cell is related to its output current^10^, which can be expressed as follows.

13 $$\:m_{{H_{2} }} = \frac{{M_{{H_{2} }} }}{{2F}}.I_{{fc}}$$

Where$\:{m}_{{H}_{2}}$is the hydrogen flow rate ($\:\frac{kg}{s}$or $\:\frac{lit}{min}$), $\:{M}_{{H}_{2}}$is molar mass of hydrogen, F is the faraday constant, $\:{I}_{fc}$is fuel cell current through the oxidizing reaction between hydrogen and oxygen. The following data could be received from Fig. 10:

- $\:{\bar{m}}^{{ON}} _{{H_{2} }}$= The average of fuel consumption in period of FTP-72 driving cycle that the PV has been used (lit/min).
- $\:{{\bar{m}}^{OFF}}_{{H}_{2}}$= The average of fuel consumption in period of FTP-72 driving cycle that the PV has not been used (lit/min).

14 $$\:{{\bar{m}}^{ON}}_{{H}_{2}}=\text{47.0475}\frac{lit}{min}\:\:\:\:\:\:\:$$

15 $$\:{{\bar{m}}^{OFF}}_{{H}_{2}}=\text{46.3437}\frac{lit}{min}\:\:\:\:\:\:$$

16 $$\:\left({{\bar{m}}^{ON}}_{{H}_{2}}\right)-\left({{\bar{m}}^{OFF}}_{{H}_{2}}\right)=\text{0.7038}\frac{lit}{min}\:\:\:$$

On the other hand, based on studied investigations of cost rate to hydrogen fuel in hybrid electric vehicles in European areas that is published by European Union in 2017, the amount of cost rate to hydrogen is determined by kilogram at 700 bar pressure^34^. Therefore, according to the studied in this work for consumed hydrogen gas by the FC source at a pressure of 1 bar at the rate of liters per minute, the calculations will be as follows. Since the easiest way to reduce the volume of gas at a constant temperature is to increase its pressure with the same mass. Therefore, at 700 bar pressure, hydrogen has a density of 36 $\:\frac{kg}{{m}^{3}}$, compared to 0.1090 $\:\frac{kg}{{m}^{3}}$under 1.5 bar. It is supposed that every gas tank of 4.7 kg hydrogen at 700 bar is equal to 270 miles driving, for example Toyota Mirai HEV. So:

17 $$\:13.72litH_{2} \times \:\frac{{1m^{3} }}{{1000lit}} = 0.01372m^{3} H_{2} \forall \:1.5bar\:\:\:\:\:\:\:\:$$

18 $$\:0.01372m^{3} H_{2} \times \:0.1090\frac{{kg}}{{m^{3} }}H_{2} = 0.001496~kgH_{2} \forall \:1.5~bar\left( {\forall \:700~bar} \right)\:\:$$

19 $$\:\left( {0.001496~kgH_{2} \times \:270mile} \right)/4.7~kgH_{2} \simeq \:0.0859miles\:\:\:\:\:\:\:\:\:\:$$

The Eq. (18) expresses that the electric propulsion of FCHEV can drive vehicle more 0.0859 miles by help of PV source in a driving road with 270miles. According to information of selected driving cycle (FTP-72) and above equations, some data could be obtained in Table 13, and also that is extracted from NEDC driving cycle with the same calculations^35^. In addition, there is a rise of extra 4.6 cycles per month if the number of driving cycles would be traveled 20 times per day.

**Table 13:** Conclusion data from study of fuel saving.

Specifications	FTP-72	NEDC
Total distance (mile)	7.45	6.85
Minimum sunlight days per month to driving (day)	20	20
Driving time of driving cycle (min)	19.6	15.65
Reduction rate of fuel consumption in period of driving cycle (%)	1.495	1.683
The amount of fuel savings in period of driving cycle per day (lit)	13.72	12.58
The amount of fuel savings in period of driving cycle per month (lit)	274.4	251.6
Monthly rate of distance efficiency traveled by 1 driving cycle per day (mile)	+ 1.718	+ 1.575
Monthly rate of distance efficiency traveled 20 driving cycles per day (mile)	+ 34.36	+ 31.50

## Conclusion

In this study, an improved hybrid electric power management algorithm for power flow between FCs, photovoltaics, batteries and SCs for EV applications was presented. The main advantage of the proposed control algorithm is the fact that it allows energy management of the vehicle without changing the algorithm. Also, the use of photovoltaic source for the first time as a relatively sustainable energy source was done alongside the FC / battery / SC in vehicle applications. The use of low-power PV in the application of a passenger car with a kW range of energy sources requires the use of a high-step up a power converter, which was implemented with the MPPT algorithm. The simulation results for two standard driving cycles of FTP-72 and NEDC to claim the capability of the proposed scheme for energy management between the sources and vehicle required power are shown in this work. Also, it is obtained around %1.5 and 1.7 of equal fuel saving for two FTP-72 and NEDC driving cycles respectively that affected by using of PV source along other electric sources. In addition, the proposed system by PV supplied is validated for some taxies which are traveled special routes (FTP-72) for many times per day by fuel saving of 4.6 driving cycles per month. This management algorithm can also be developed for other topologies of power converters (isolated or interleaved) and adding energy sources such as wind turbines (in the front bumper) and or flywheel.

## Electronic supplementary material

Below is the link to the electronic supplementary material.

Supplementary Material 1

## Data Availability

Data is provided as a supplementary file. We declare that any available data will be provided.

## List of symbols

$$\:{M}_{V}$$: Vehicle Mas
$\:\delta\:$: Mass coefficient of rotary elements
$$\:{C}_{D}$$: Drag coefficient
$\:{f}_{r}$: Rolling resistance coefficient
g: Field of gravity
*i*: Road grade (the ability of vehicle to pass of slopes)
$$\:{\rho\:}_{a}$$: Air density
$$\:\alpha\:$$: Road angle
$\:{A}_{f}$: Vehicle front area
$\:{\eta\:}_{em}$: Efficiency of electric motor
$\:{\eta\:}_{t}$: Efficiency of transmission system
$\:{\eta\:}_{m}$: Efficiency of machine
$\:{P}_{aux}$: Auxiliary power for automobile
$\:{K}_{V}$: PV Voltage Temperature Coefficient (%)
$\:{K}_{i}\:$: PV Current Temperature Coefficient (%)
$\:{C}_{p}$: The percentage of total energy capacity of supercapacitor (is allowed to be used)
$\:{V}_{DC}$: DC voltage of common bus
$\:{V}_{pv}\:$: Output voltage of the solar panel
$\:{X}^{*}\:$: Position vector of the best solution obtained
$\:{k}_{p}\:$: Proportional parameter in Fractional Order Proportional Integral Controller
$\:{k}_{I}\:$: Integral parameter in Fractional Order Proportional Integral Controller
$\:\lambda\:\:$: Rank parameter in Fractional Order Proportional Integral Controller
F: Faraday constant
$\:{m}_{{H}_{2}}$: Hydrogen flow rate
$\:{M}_{{H}_{2}}$: Hydrogen molar mass

## Abbreviations

AC: Alternate Current
DC: Direct Current
EV: Electric Vehicle
FC: Fuel Cell
SC: Super Capacitor
PV: Photovoltaic
PEMFC: Proton Exchange Membrane Fuel cell
FOPI: Fractional Order Proportional Integral
WOA: Whale Optimization Algorithm
EMA: Energy Management Algorithm
