# Supplementary material for: A comprehensive scheme for power management of FC/SC/battery, and solar-roof PV source in electric vehicle systems
Source: Sci Rep. 2024 Nov 11;14:27621. doi: 10.1038/s41598-024-79241-1 (PMC11555224; doi:10.1038/s41598-024-79241-1)
Supplement: Supplementary file 1 — Supplementary Material 1 [file 41598_2024_79241_MOESM1_ESM.docx]

The Urban Dynamometer Driving Schedule is a mandated dynamometer test on tailpipe emissions of a car that represents city driving conditions.

It is also known as FTP-72 or LA-4, and it is also used in Sweden as the A10 or CVS (Constant Volume Sampler) cycle and in Australia as the ADR 27 (Australian Design Rules) cycle.

The cycle simulates an urban route of 12.07 km (7.5 mi) with frequent stops. The maximum speed is 91.2 km/h (56.7 mi/h) and the average speed is 31.5 km/h (19.6 mi/h).

The cycle has two phases: a "cold start" phase of 505 seconds over a projected distance of 5.78 km at 41.2 km/h average speed, and a "transient phase" of 864 seconds, for a total duration of 1369 seconds. The first two phases are run together as one large phase and followed by stopping the engine for 10 minutes, then restarting the engine to test warm restart, and re-running the first phase again (505 seconds) to make the complete 3 phase FTP-75 {July 2014}. In the U.S., weighting factors are applied to the emissions constituent numbers to add a correction for the frequency of how often a typical cycle would be performed in the real world. The first phase (cold start, 505 seconds) has a correction of 0.43 as cold starting is encountered less often. Phase 2 (864 seconds of city drive) has a weighting of 1.0, meaning the constituents in this area are counted full to stress the need for cleanliness in normal warmed-up drive, and the 0.57 is applied to the third phase (warm start, 505 seconds, repeat of phase one only with a warm engine start, which indicates that the real-world effect of warm starts happen more often than cold starts.

| FTPCOL.TXT | Federal Test Procedure |
| --- | --- |
| Test Time (sec) | Target Speed (mil/h) |
| 0 | 0 |
| 1 | 0 |
| 2 | 0 |
| 3 | 0 |
| 4 | 0 |
| 5 | 0 |
| 6 | 0 |
| 7 | 0 |
| 8 | 0 |
| 9 | 0 |
| 10 | 0 |
| 11 | 0 |
| 12 | 0 |
| 13 | 0 |
| 14 | 0 |
| 15 | 0 |
| 16 | 0 |
| 17 | 0 |
| 18 | 0 |
| 19 | 0 |
| 20 | 0 |
| 21 | 3 |
| 22 | 5.9 |
| 23 | 8.6 |
| 24 | 11.5 |
| 25 | 14.3 |
| 26 | 16.9 |
| 27 | 17.3 |
| 28 | 18.1 |
| 29 | 20.7 |
| 30 | 21.7 |
| 31 | 22.4 |
| 32 | 22.5 |
| 33 | 22.1 |
| 34 | 21.5 |
| 35 | 20.9 |
| 36 | 20.4 |
| 37 | 19.8 |
| 38 | 17 |
| 39 | 14.9 |
| 40 | 14.9 |
| 41 | 15.2 |
| 42 | 15.5 |
| 43 | 16 |
| 44 | 17.1 |
| 45 | 19.1 |
| 46 | 21.1 |
| 47 | 22.7 |
| 48 | 22.9 |
| 49 | 22.7 |
| 50 | 22.6 |
| 51 | 21.3 |
| 52 | 19 |
| 53 | 17.1 |
| 54 | 15.8 |
| 55 | 15.8 |
| 56 | 17.7 |
| 57 | 19.8 |
| 58 | 21.6 |
| 59 | 23.2 |
| 60 | 24.2 |
| 61 | 24.6 |
| 62 | 24.9 |
| 63 | 25 |
| 64 | 24.6 |
| 65 | 24.5 |
| 66 | 24.7 |
| 67 | 24.8 |
| 68 | 24.7 |
| 69 | 24.6 |
| 70 | 24.6 |
| 71 | 25.1 |
| 72 | 25.6 |
| 73 | 25.7 |
| 74 | 25.4 |
| 75 | 24.9 |
| 76 | 25 |
| 77 | 25.4 |
| 78 | 26 |
| 79 | 26 |
| 80 | 25.7 |
| 81 | 26.1 |
| 82 | 26.7 |
| 83 | 27.5 |
| 84 | 28.6 |
| 85 | 29.3 |
| 86 | 29.8 |
| 87 | 30.1 |
| 88 | 30.4 |
| 89 | 30.7 |
| 90 | 30.7 |
| 91 | 30.5 |
| 92 | 30.4 |
| 93 | 30.3 |
| 94 | 30.4 |
| 95 | 30.8 |
| 96 | 30.4 |
| 97 | 29.9 |
| 98 | 29.5 |
| 99 | 29.8 |
| 100 | 30.3 |
| 101 | 30.7 |
| 102 | 30.9 |
| 103 | 31 |
| 104 | 30.9 |
| 105 | 30.4 |
| 106 | 29.8 |
| 107 | 29.9 |
| 108 | 30.2 |
| 109 | 30.7 |
| 110 | 31.2 |
| 111 | 31.8 |
| 112 | 32.2 |
| 113 | 32.4 |
| 114 | 32.2 |
| 115 | 31.7 |
| 116 | 28.6 |
| 117 | 25.3 |
| 118 | 22 |
| 119 | 18.7 |
| 120 | 15.4 |
| 121 | 12.1 |
| 122 | 8.8 |
| 123 | 5.5 |
| 124 | 2.2 |
| 125 | 0 |
| 126 | 0 |
| 127 | 0 |
| 128 | 0 |
| 129 | 0 |
| 130 | 0 |
| 131 | 0 |
| 132 | 0 |
| 133 | 0 |
| 134 | 0 |
| 135 | 0 |
| 136 | 0 |
| 137 | 0 |
| 138 | 0 |
| 139 | 0 |
| 140 | 0 |
| 141 | 0 |
| 142 | 0 |
| 143 | 0 |
| 144 | 0 |
| 145 | 0 |
| 146 | 0 |
| 147 | 0 |
| 148 | 0 |
| 149 | 0 |
| 150 | 0 |
| 151 | 0 |
| 152 | 0 |
| 153 | 0 |
| 154 | 0 |
| 155 | 0 |
| 156 | 0 |
| 157 | 0 |
| 158 | 0 |
| 159 | 0 |
| 160 | 0 |
| 161 | 0 |
| 162 | 0 |
| 163 | 0 |
| 164 | 3.3 |
| 165 | 6.6 |
| 166 | 9.9 |
| 167 | 13.2 |
| 168 | 16.5 |
| 169 | 19.8 |
| 170 | 22.2 |
| 171 | 24.3 |
| 172 | 25.8 |
| 173 | 26.4 |
| 174 | 25.7 |
| 175 | 25.1 |
| 176 | 24.7 |
| 177 | 25 |
| 178 | 25.2 |
| 179 | 25.4 |
| 180 | 25.8 |
| 181 | 27.2 |
| 182 | 26.5 |
| 183 | 24 |
| 184 | 22.7 |
| 185 | 19.4 |
| 186 | 17.7 |
| 187 | 17.2 |
| 188 | 18.1 |
| 189 | 18.6 |
| 190 | 20 |
| 191 | 22.2 |
| 192 | 24.5 |
| 193 | 27.3 |
| 194 | 30.5 |
| 195 | 33.5 |
| 196 | 36.2 |
| 197 | 37.3 |
| 198 | 39.3 |
| 199 | 40.5 |
| 200 | 42.1 |
| 201 | 43.5 |
| 202 | 45.1 |
| 203 | 46 |
| 204 | 46.8 |
| 205 | 47.5 |
| 206 | 47.5 |
| 207 | 47.3 |
| 208 | 47.2 |
| 209 | 47 |
| 210 | 47 |
| 211 | 47 |
| 212 | 47 |
| 213 | 47 |
| 214 | 47.2 |
| 215 | 47.4 |
| 216 | 47.9 |
| 217 | 48.5 |
| 218 | 49.1 |
| 219 | 49.5 |
| 220 | 50 |
| 221 | 50.6 |
| 222 | 51 |
| 223 | 51.5 |
| 224 | 52.2 |
| 225 | 53.2 |
| 226 | 54.1 |
| 227 | 54.6 |
| 228 | 54.9 |
| 229 | 55 |
| 230 | 54.9 |
| 231 | 54.6 |
| 232 | 54.6 |
| 233 | 54.8 |
| 234 | 55.1 |
| 235 | 55.5 |
| 236 | 55.7 |
| 237 | 56.1 |
| 238 | 56.3 |
| 239 | 56.6 |
| 240 | 56.7 |
| 241 | 56.7 |
| 242 | 56.5 |
| 243 | 56.5 |
| 244 | 56.5 |
| 245 | 56.5 |
| 246 | 56.5 |
| 247 | 56.5 |
| 248 | 56.4 |
| 249 | 56.1 |
| 250 | 55.8 |
| 251 | 55.1 |
| 252 | 54.6 |
| 253 | 54.2 |
| 254 | 54 |
| 255 | 53.7 |
| 256 | 53.6 |
| 257 | 53.9 |
| 258 | 54 |
| 259 | 54.1 |
| 260 | 54.1 |
| 261 | 53.8 |
| 262 | 53.4 |
| 263 | 53 |
| 264 | 52.6 |
| 265 | 52.1 |
| 266 | 52.4 |
| 267 | 52 |
| 268 | 51.9 |
| 269 | 51.7 |
| 270 | 51.5 |
| 271 | 51.6 |
| 272 | 51.8 |
| 273 | 52.1 |
| 274 | 52.5 |
| 275 | 53 |
| 276 | 53.5 |
| 277 | 54 |
| 278 | 54.9 |
| 279 | 55.4 |
| 280 | 55.6 |
| 281 | 56 |
| 282 | 56 |
| 283 | 55.8 |
| 284 | 55.2 |
| 285 | 54.5 |
| 286 | 53.6 |
| 287 | 52.5 |
| 288 | 51.5 |
| 289 | 51.5 |
| 290 | 51.5 |
| 291 | 51.1 |
| 292 | 50.1 |
| 293 | 50 |
| 294 | 50.1 |
| 295 | 50 |
| 296 | 49.6 |
| 297 | 49.5 |
| 298 | 49.5 |
| 299 | 49.5 |
| 300 | 49.1 |
| 301 | 48.6 |
| 302 | 48.1 |
| 303 | 47.2 |
| 304 | 46.1 |
| 305 | 45 |
| 306 | 43.8 |
| 307 | 42.6 |
| 308 | 41.5 |
| 309 | 40.3 |
| 310 | 38.5 |
| 311 | 37 |
| 312 | 35.2 |
| 313 | 33.8 |
| 314 | 32.5 |
| 315 | 31.5 |
| 316 | 30.6 |
| 317 | 30.5 |
| 318 | 30 |
| 319 | 29 |
| 320 | 27.5 |
| 321 | 24.8 |
| 322 | 21.5 |
| 323 | 20.1 |
| 324 | 19.1 |
| 325 | 18.5 |
| 326 | 17 |
| 327 | 15.5 |
| 328 | 12.5 |
| 329 | 10.8 |
| 330 | 8 |
| 331 | 4.7 |
| 332 | 1.4 |
| 333 | 0 |
| 334 | 0 |
| 335 | 0 |
| 336 | 0 |
| 337 | 0 |
| 338 | 0 |
| 339 | 0 |
| 340 | 0 |
| 341 | 0 |
| 342 | 0 |
| 343 | 0 |
| 344 | 0 |
| 345 | 0 |
| 346 | 0 |
| 347 | 1 |
| 348 | 4.3 |
| 349 | 7.6 |
| 350 | 10.9 |
| 351 | 14.2 |
| 352 | 17.3 |
| 353 | 20 |
| 354 | 22.5 |
| 355 | 23.7 |
| 356 | 25.2 |
| 357 | 26.6 |
| 358 | 28.1 |
| 359 | 30 |
| 360 | 30.8 |
| 361 | 31.6 |
| 362 | 32.1 |
| 363 | 32.8 |
| 364 | 33.6 |
| 365 | 34.5 |
| 366 | 34.6 |
| 367 | 34.9 |
| 368 | 34.8 |
| 369 | 34.5 |
| 370 | 34.7 |
| 371 | 35.5 |
| 372 | 36 |
| 373 | 36 |
| 374 | 36 |
| 375 | 36 |
| 376 | 36 |
| 377 | 36 |
| 378 | 36.1 |
| 379 | 36.4 |
| 380 | 36.5 |
| 381 | 36.4 |
| 382 | 36 |
| 383 | 35.1 |
| 384 | 34.1 |
| 385 | 33.5 |
| 386 | 31.4 |
| 387 | 29 |
| 388 | 25.7 |
| 389 | 23 |
| 390 | 20.3 |
| 391 | 17.5 |
| 392 | 14.5 |
| 393 | 12 |
| 394 | 8.7 |
| 395 | 5.4 |
| 396 | 2.1 |
| 397 | 0 |
| 398 | 0 |
| 399 | 0 |
| 400 | 0 |
| 401 | 0 |
| 402 | 0 |
| 403 | 2.6 |
| 404 | 5.9 |
| 405 | 9.2 |
| 406 | 12.5 |
| 407 | 15.8 |
| 408 | 19.1 |
| 409 | 22.4 |
| 410 | 25 |
| 411 | 25.6 |
| 412 | 27.5 |
| 413 | 29 |
| 414 | 30 |
| 415 | 30.1 |
| 416 | 30 |
| 417 | 29.7 |
| 418 | 29.3 |
| 419 | 28.8 |
| 420 | 28 |
| 421 | 25 |
| 422 | 21.7 |
| 423 | 18.4 |
| 424 | 15.1 |
| 425 | 11.8 |
| 426 | 8.5 |
| 427 | 5.2 |
| 428 | 1.9 |
| 429 | 0 |
| 430 | 0 |
| 431 | 0 |
| 432 | 0 |
| 433 | 0 |
| 434 | 0 |
| 435 | 0 |
| 436 | 0 |
| 437 | 0 |
| 438 | 0 |
| 439 | 0 |
| 440 | 0 |
| 441 | 0 |
| 442 | 0 |
| 443 | 0 |
| 444 | 0 |
| 445 | 0 |
| 446 | 0 |
| 447 | 0 |
| 448 | 3.3 |
| 449 | 6.6 |
| 450 | 9.9 |
| 451 | 13.2 |
| 452 | 16.5 |
| 453 | 19.8 |
| 454 | 23.1 |
| 455 | 26.4 |
| 456 | 27.8 |
| 457 | 29.1 |
| 458 | 31.5 |
| 459 | 33 |
| 460 | 33.6 |
| 461 | 34.8 |
| 462 | 35.1 |
| 463 | 35.6 |
| 464 | 36.1 |
| 465 | 36 |
| 466 | 36.1 |
| 467 | 36.2 |
| 468 | 36 |
| 469 | 35.7 |
| 470 | 36 |
| 471 | 36 |
| 472 | 35.6 |
| 473 | 35.5 |
| 474 | 35.4 |
| 475 | 35.2 |
| 476 | 35.2 |
| 477 | 35.2 |
| 478 | 35.2 |
| 479 | 35.2 |
| 480 | 35.2 |
| 481 | 35 |
| 482 | 35.1 |
| 483 | 35.2 |
| 484 | 35.5 |
| 485 | 35.2 |
| 486 | 35 |
| 487 | 35 |
| 488 | 35 |
| 489 | 34.8 |
| 490 | 34.6 |
| 491 | 34.5 |
| 492 | 33.5 |
| 493 | 32 |
| 494 | 30.1 |
| 495 | 28 |
| 496 | 25.5 |
| 497 | 22.5 |
| 498 | 19.8 |
| 499 | 16.5 |
| 500 | 13.2 |
| 501 | 10.3 |
| 502 | 7.2 |
| 503 | 4 |
| 504 | 1 |
| 505 | 0 |
| 506 | 0 |
| 507 | 0 |
| 508 | 0 |
| 509 | 0 |
| 510 | 0 |
| 511 | 1.2 |
| 512 | 3.5 |
| 513 | 5.5 |
| 514 | 6.5 |
| 515 | 8.5 |
| 516 | 9.6 |
| 517 | 10.5 |
| 518 | 11.9 |
| 519 | 14 |
| 520 | 16 |
| 521 | 17.7 |
| 522 | 19 |
| 523 | 20.1 |
| 524 | 21 |
| 525 | 22 |
| 526 | 23 |
| 527 | 23.8 |
| 528 | 24.5 |
| 529 | 24.9 |
| 530 | 25 |
| 531 | 25 |
| 532 | 25 |
| 533 | 25 |
| 534 | 25 |
| 535 | 25 |
| 536 | 25.6 |
| 537 | 25.8 |
| 538 | 26 |
| 539 | 25.6 |
| 540 | 25.2 |
| 541 | 25 |
| 542 | 25 |
| 543 | 25 |
| 544 | 24.4 |
| 545 | 23.1 |
| 546 | 19.8 |
| 547 | 16.5 |
| 548 | 13.2 |
| 549 | 9.9 |
| 550 | 6.6 |
| 551 | 3.3 |
| 552 | 0 |
| 553 | 0 |
| 554 | 0 |
| 555 | 0 |
| 556 | 0 |
| 557 | 0 |
| 558 | 0 |
| 559 | 0 |
| 560 | 0 |
| 561 | 0 |
| 562 | 0 |
| 563 | 0 |
| 564 | 0 |
| 565 | 0 |
| 566 | 0 |
| 567 | 0 |
| 568 | 0 |
| 569 | 3.3 |
| 570 | 6.6 |
| 571 | 9.9 |
| 572 | 13 |
| 573 | 14.6 |
| 574 | 16 |
| 575 | 17 |
| 576 | 17 |
| 577 | 17 |
| 578 | 17.5 |
| 579 | 17.7 |
| 580 | 17.7 |
| 581 | 17.5 |
| 582 | 17 |
| 583 | 16.9 |
| 584 | 16.6 |
| 585 | 17 |
| 586 | 17.1 |
| 587 | 17 |
| 588 | 16.6 |
| 589 | 16.5 |
| 590 | 16.5 |
| 591 | 16.6 |
| 592 | 17 |
| 593 | 17.6 |
| 594 | 18.5 |
| 595 | 19.2 |
| 596 | 20.2 |
| 597 | 21 |
| 598 | 21.1 |
| 599 | 21.2 |
| 600 | 21.6 |
| 601 | 22 |
| 602 | 22.4 |
| 603 | 22.5 |
| 604 | 22.5 |
| 605 | 22.5 |
| 606 | 22.7 |
| 607 | 23.7 |
| 608 | 25.1 |
| 609 | 26 |
| 610 | 26.5 |
| 611 | 27 |
| 612 | 26.1 |
| 613 | 22.8 |
| 614 | 19.5 |
| 615 | 16.2 |
| 616 | 12.9 |
| 617 | 9.6 |
| 618 | 6.3 |
| 619 | 3 |
| 620 | 0 |
| 621 | 0 |
| 622 | 0 |
| 623 | 0 |
| 624 | 0 |
| 625 | 0 |
| 626 | 0 |
| 627 | 0 |
| 628 | 0 |
| 629 | 0 |
| 630 | 0 |
| 631 | 0 |
| 632 | 0 |
| 633 | 0 |
| 634 | 0 |
| 635 | 0 |
| 636 | 0 |
| 637 | 0 |
| 638 | 0 |
| 639 | 0 |
| 640 | 0 |
| 641 | 0 |
| 642 | 0 |
| 643 | 0 |
| 644 | 0 |
| 645 | 0 |
| 646 | 2 |
| 647 | 4.5 |
| 648 | 7.8 |
| 649 | 10.2 |
| 650 | 12.5 |
| 651 | 14 |
| 652 | 15.3 |
| 653 | 17.5 |
| 654 | 19.6 |
| 655 | 21 |
| 656 | 22.2 |
| 657 | 23.3 |
| 658 | 24.5 |
| 659 | 25.3 |
| 660 | 25.6 |
| 661 | 26 |
| 662 | 26.1 |
| 663 | 26.2 |
| 664 | 26.2 |
| 665 | 26.4 |
| 666 | 26.5 |
| 667 | 26.5 |
| 668 | 26 |
| 669 | 25.5 |
| 670 | 23.6 |
| 671 | 21.4 |
| 672 | 18.5 |
| 673 | 16.4 |
| 674 | 14.5 |
| 675 | 11.6 |
| 676 | 8.7 |
| 677 | 5.8 |
| 678 | 3.5 |
| 679 | 2 |
| 680 | 0 |
| 681 | 0 |
| 682 | 0 |
| 683 | 0 |
| 684 | 0 |
| 685 | 0 |
| 686 | 0 |
| 687 | 0 |
| 688 | 0 |
| 689 | 0 |
| 690 | 0 |
| 691 | 0 |
| 692 | 0 |
| 693 | 0 |
| 694 | 1.4 |
| 695 | 3.3 |
| 696 | 4.4 |
| 697 | 6.5 |
| 698 | 9.2 |
| 699 | 11.3 |
| 700 | 13.5 |
| 701 | 14.6 |
| 702 | 16.4 |
| 703 | 16.7 |
| 704 | 16.5 |
| 705 | 16.5 |
| 706 | 18.2 |
| 707 | 19.2 |
| 708 | 20.1 |
| 709 | 21.5 |
| 710 | 22.5 |
| 711 | 22.5 |
| 712 | 22.1 |
| 713 | 22.7 |
| 714 | 23.3 |
| 715 | 23.5 |
| 716 | 22.5 |
| 717 | 21.6 |
| 718 | 20.5 |
| 719 | 18 |
| 720 | 15 |
| 721 | 12 |
| 722 | 9 |
| 723 | 6.2 |
| 724 | 4.5 |
| 725 | 3 |
| 726 | 2.1 |
| 727 | 0.5 |
| 728 | 0.5 |
| 729 | 3.2 |
| 730 | 6.5 |
| 731 | 9.6 |
| 732 | 12.5 |
| 733 | 14 |
| 734 | 16 |
| 735 | 18 |
| 736 | 19.6 |
| 737 | 21.5 |
| 738 | 23.1 |
| 739 | 24.5 |
| 740 | 25.5 |
| 741 | 26.5 |
| 742 | 27.1 |
| 743 | 27.6 |
| 744 | 27.9 |
| 745 | 28.3 |
| 746 | 28.6 |
| 747 | 28.6 |
| 748 | 28.3 |
| 749 | 28.2 |
| 750 | 28 |
| 751 | 27.5 |
| 752 | 26.8 |
| 753 | 25.5 |
| 754 | 23.5 |
| 755 | 21.5 |
| 756 | 19 |
| 757 | 16.5 |
| 758 | 14.9 |
| 759 | 12.5 |
| 760 | 9.4 |
| 761 | 6.2 |
| 762 | 3 |
| 763 | 1.5 |
| 764 | 1.5 |
| 765 | 0.5 |
| 766 | 0 |
| 767 | 3 |
| 768 | 6.3 |
| 769 | 9.6 |
| 770 | 12.9 |
| 771 | 15.8 |
| 772 | 17.5 |
| 773 | 18.4 |
| 774 | 19.5 |
| 775 | 20.7 |
| 776 | 22 |
| 777 | 23.2 |
| 778 | 25 |
| 779 | 26.5 |
| 780 | 27.5 |
| 781 | 28 |
| 782 | 28.3 |
| 783 | 28.9 |
| 784 | 28.9 |
| 785 | 28.9 |
| 786 | 28.8 |
| 787 | 28.5 |
| 788 | 28.3 |
| 789 | 28.3 |
| 790 | 28.3 |
| 791 | 28.2 |
| 792 | 27.6 |
| 793 | 27.5 |
| 794 | 27.5 |
| 795 | 27.5 |
| 796 | 27.5 |
| 797 | 27.5 |
| 798 | 27.5 |
| 799 | 27.6 |
| 800 | 28 |
| 801 | 28.5 |
| 802 | 30 |
| 803 | 31 |
| 804 | 32 |
| 805 | 33 |
| 806 | 33 |
| 807 | 33.6 |
| 808 | 34 |
| 809 | 34.3 |
| 810 | 34.2 |
| 811 | 34 |
| 812 | 34 |
| 813 | 33.9 |
| 814 | 33.6 |
| 815 | 33.1 |
| 816 | 33 |
| 817 | 32.5 |
| 818 | 32 |
| 819 | 31.9 |
| 820 | 31.6 |
| 821 | 31.5 |
| 822 | 30.6 |
| 823 | 30 |
| 824 | 29.9 |
| 825 | 29.9 |
| 826 | 29.9 |
| 827 | 29.9 |
| 828 | 29.6 |
| 829 | 29.5 |
| 830 | 29.5 |
| 831 | 29.3 |
| 832 | 28.9 |
| 833 | 28.2 |
| 834 | 27.7 |
| 835 | 27 |
| 836 | 25.5 |
| 837 | 23.7 |
| 838 | 22 |
| 839 | 20.5 |
| 840 | 19.2 |
| 841 | 19.2 |
| 842 | 20.1 |
| 843 | 20.9 |
| 844 | 21.4 |
| 845 | 22 |
| 846 | 22.6 |
| 847 | 23.2 |
| 848 | 24 |
| 849 | 25 |
| 850 | 26 |
| 851 | 26.6 |
| 852 | 26.6 |
| 853 | 26.8 |
| 854 | 27 |
| 855 | 27.2 |
| 856 | 27.8 |
| 857 | 28.1 |
| 858 | 28.8 |
| 859 | 28.9 |
| 860 | 29 |
| 861 | 29.1 |
| 862 | 29 |
| 863 | 28.1 |
| 864 | 27.5 |
| 865 | 27 |
| 866 | 25.8 |
| 867 | 25 |
| 868 | 24.5 |
| 869 | 24.8 |
| 870 | 25.1 |
| 871 | 25.5 |
| 872 | 25.7 |
| 873 | 26.2 |
| 874 | 26.9 |
| 875 | 27.5 |
| 876 | 27.8 |
| 877 | 28.4 |
| 878 | 29 |
| 879 | 29.2 |
| 880 | 29.1 |
| 881 | 29 |
| 882 | 28.9 |
| 883 | 28.5 |
| 884 | 28.1 |
| 885 | 28 |
| 886 | 28 |
| 887 | 27.6 |
| 888 | 27.2 |
| 889 | 26.6 |
| 890 | 27 |
| 891 | 27.5 |
| 892 | 27.8 |
| 893 | 28 |
| 894 | 27.8 |
| 895 | 28 |
| 896 | 28 |
| 897 | 28 |
| 898 | 27.7 |
| 899 | 27.4 |
| 900 | 26.9 |
| 901 | 26.6 |
| 902 | 26.5 |
| 903 | 26.5 |
| 904 | 26.5 |
| 905 | 26.3 |
| 906 | 26.2 |
| 907 | 26.2 |
| 908 | 25.9 |
| 909 | 25.6 |
| 910 | 25.6 |
| 911 | 25.9 |
| 912 | 25.8 |
| 913 | 25.5 |
| 914 | 24.6 |
| 915 | 23.5 |
| 916 | 22.2 |
| 917 | 21.6 |
| 918 | 21.6 |
| 919 | 21.7 |
| 920 | 22.6 |
| 921 | 23.4 |
| 922 | 24 |
| 923 | 24.2 |
| 924 | 24.4 |
| 925 | 24.9 |
| 926 | 25.1 |
| 927 | 25.2 |
| 928 | 25.3 |
| 929 | 25.5 |
| 930 | 25.2 |
| 931 | 25 |
| 932 | 25 |
| 933 | 25 |
| 934 | 24.7 |
| 935 | 24.5 |
| 936 | 24.3 |
| 937 | 24.3 |
| 938 | 24.5 |
| 939 | 25 |
| 940 | 25 |
| 941 | 24.6 |
| 942 | 24.6 |
| 943 | 24.1 |
| 944 | 24.5 |
| 945 | 25.1 |
| 946 | 25.6 |
| 947 | 25.1 |
| 948 | 24 |
| 949 | 22 |
| 950 | 20.1 |
| 951 | 16.9 |
| 952 | 13.6 |
| 953 | 10.3 |
| 954 | 7 |
| 955 | 3.7 |
| 956 | 0.4 |
| 957 | 0 |
| 958 | 0 |
| 959 | 0 |
| 960 | 2 |
| 961 | 5.3 |
| 962 | 8.6 |
| 963 | 11.9 |
| 964 | 15.2 |
| 965 | 17.5 |
| 966 | 18.6 |
| 967 | 20 |
| 968 | 21.1 |
| 969 | 22 |
| 970 | 23 |
| 971 | 24.5 |
| 972 | 26.3 |
| 973 | 27.5 |
| 974 | 28.1 |
| 975 | 28.4 |
| 976 | 28.5 |
| 977 | 28.5 |
| 978 | 28.5 |
| 979 | 27.7 |
| 980 | 27.5 |
| 981 | 27.2 |
| 982 | 26.8 |
| 983 | 26.5 |
| 984 | 26 |
| 985 | 25.7 |
| 986 | 25.2 |
| 987 | 24 |
| 988 | 22 |
| 989 | 21.5 |
| 990 | 21.5 |
| 991 | 21.8 |
| 992 | 22.5 |
| 993 | 23 |
| 994 | 22.8 |
| 995 | 22.8 |
| 996 | 23 |
| 997 | 22.7 |
| 998 | 22.7 |
| 999 | 22.7 |
| 1000 | 23.5 |
| 1001 | 24 |
| 1002 | 24.6 |
| 1003 | 24.8 |
| 1004 | 25.1 |
| 1005 | 25.5 |
| 1006 | 25.6 |
| 1007 | 25.5 |
| 1008 | 25 |
| 1009 | 24.1 |
| 1010 | 23.7 |
| 1011 | 23.2 |
| 1012 | 22.9 |
| 1013 | 22.5 |
| 1014 | 22 |
| 1015 | 21.6 |
| 1016 | 20.5 |
| 1017 | 17.5 |
| 1018 | 14.2 |
| 1019 | 10.9 |
| 1020 | 7.6 |
| 1021 | 4.3 |
| 1022 | 1 |
| 1023 | 0 |
| 1024 | 0 |
| 1025 | 0 |
| 1026 | 0 |
| 1027 | 0 |
| 1028 | 0 |
| 1029 | 0 |
| 1030 | 0 |
| 1031 | 0 |
| 1032 | 0 |
| 1033 | 0 |
| 1034 | 0 |
| 1035 | 0 |
| 1036 | 0 |
| 1037 | 0 |
| 1038 | 0 |
| 1039 | 0 |
| 1040 | 0 |
| 1041 | 0 |
| 1042 | 0 |
| 1043 | 0 |
| 1044 | 0 |
| 1045 | 0 |
| 1046 | 0 |
| 1047 | 0 |
| 1048 | 0 |
| 1049 | 0 |
| 1050 | 0 |
| 1051 | 0 |
| 1052 | 0 |
| 1053 | 1.2 |
| 1054 | 4 |
| 1055 | 7.3 |
| 1056 | 10.6 |
| 1057 | 13.9 |
| 1058 | 17 |
| 1059 | 18.5 |
| 1060 | 20 |
| 1061 | 21.8 |
| 1062 | 23 |
| 1063 | 24 |
| 1064 | 24.8 |
| 1065 | 25.6 |
| 1066 | 26.5 |
| 1067 | 26.8 |
| 1068 | 27.4 |
| 1069 | 27.9 |
| 1070 | 28.3 |
| 1071 | 28 |
| 1072 | 27.5 |
| 1073 | 27 |
| 1074 | 27 |
| 1075 | 26.3 |
| 1076 | 24.5 |
| 1077 | 22.5 |
| 1078 | 21.5 |
| 1079 | 20.6 |
| 1080 | 18 |
| 1081 | 15 |
| 1082 | 12.3 |
| 1083 | 11.1 |
| 1084 | 10.6 |
| 1085 | 10 |
| 1086 | 9.5 |
| 1087 | 9.1 |
| 1088 | 8.7 |
| 1089 | 8.6 |
| 1090 | 8.8 |
| 1091 | 9 |
| 1092 | 8.7 |
| 1093 | 8.6 |
| 1094 | 8 |
| 1095 | 7 |
| 1096 | 5 |
| 1097 | 4.2 |
| 1098 | 2.6 |
| 1099 | 1 |
| 1100 | 0 |
| 1101 | 0.1 |
| 1102 | 0.6 |
| 1103 | 1.6 |
| 1104 | 3.6 |
| 1105 | 6.9 |
| 1106 | 10 |
| 1107 | 12.8 |
| 1108 | 14 |
| 1109 | 14.5 |
| 1110 | 16 |
| 1111 | 18.1 |
| 1112 | 20 |
| 1113 | 21 |
| 1114 | 21.2 |
| 1115 | 21.3 |
| 1116 | 21.4 |
| 1117 | 21.7 |
| 1118 | 22.5 |
| 1119 | 23 |
| 1120 | 23.8 |
| 1121 | 24.5 |
| 1122 | 25 |
| 1123 | 24.9 |
| 1124 | 24.8 |
| 1125 | 25 |
| 1126 | 25.4 |
| 1127 | 25.8 |
| 1128 | 26 |
| 1129 | 26.4 |
| 1130 | 26.6 |
| 1131 | 26.9 |
| 1132 | 27 |
| 1133 | 27 |
| 1134 | 27 |
| 1135 | 26.9 |
| 1136 | 26.8 |
| 1137 | 26.8 |
| 1138 | 26.5 |
| 1139 | 26.4 |
| 1140 | 26 |
| 1141 | 25.5 |
| 1142 | 24.6 |
| 1143 | 23.5 |
| 1144 | 21.5 |
| 1145 | 20 |
| 1146 | 17.5 |
| 1147 | 16 |
| 1148 | 14 |
| 1149 | 10.7 |
| 1150 | 7.4 |
| 1151 | 4.1 |
| 1152 | 0.8 |
| 1153 | 0 |
| 1154 | 0 |
| 1155 | 0 |
| 1156 | 0 |
| 1157 | 0 |
| 1158 | 0 |
| 1159 | 0 |
| 1160 | 0 |
| 1161 | 0 |
| 1162 | 0 |
| 1163 | 0 |
| 1164 | 0 |
| 1165 | 0 |
| 1166 | 0 |
| 1167 | 0 |
| 1168 | 0 |
| 1169 | 2.1 |
| 1170 | 5.4 |
| 1171 | 8.7 |
| 1172 | 12 |
| 1173 | 15.3 |
| 1174 | 18.6 |
| 1175 | 21.1 |
| 1176 | 23 |
| 1177 | 23.5 |
| 1178 | 23 |
| 1179 | 22.5 |
| 1180 | 20 |
| 1181 | 16.7 |
| 1182 | 13.4 |
| 1183 | 10.1 |
| 1184 | 6.8 |
| 1185 | 3.5 |
| 1186 | 0.2 |
| 1187 | 0 |
| 1188 | 0 |
| 1189 | 0 |
| 1190 | 0 |
| 1191 | 0 |
| 1192 | 0 |
| 1193 | 0 |
| 1194 | 0 |
| 1195 | 0 |
| 1196 | 0 |
| 1197 | 0.2 |
| 1198 | 1.5 |
| 1199 | 3.5 |
| 1200 | 6.5 |
| 1201 | 9.8 |
| 1202 | 12 |
| 1203 | 12.9 |
| 1204 | 13 |
| 1205 | 12.6 |
| 1206 | 12.8 |
| 1207 | 13.1 |
| 1208 | 13.1 |
| 1209 | 14 |
| 1210 | 15.5 |
| 1211 | 17 |
| 1212 | 18.6 |
| 1213 | 19.7 |
| 1214 | 21 |
| 1215 | 21.5 |
| 1216 | 21.8 |
| 1217 | 21.8 |
| 1218 | 21.5 |
| 1219 | 21.2 |
| 1220 | 21.5 |
| 1221 | 21.8 |
| 1222 | 22 |
| 1223 | 21.9 |
| 1224 | 21.7 |
| 1225 | 21.5 |
| 1226 | 21.5 |
| 1227 | 21.4 |
| 1228 | 20.1 |
| 1229 | 19.5 |
| 1230 | 19.2 |
| 1231 | 19.6 |
| 1232 | 19.8 |
| 1233 | 20 |
| 1234 | 19.5 |
| 1235 | 17.5 |
| 1236 | 15.5 |
| 1237 | 13 |
| 1238 | 10 |
| 1239 | 8 |
| 1240 | 6 |
| 1241 | 4 |
| 1242 | 2.5 |
| 1243 | 0.7 |
| 1244 | 0 |
| 1245 | 0 |
| 1246 | 0 |
| 1247 | 0 |
| 1248 | 0 |
| 1249 | 0 |
| 1250 | 0 |
| 1251 | 0 |
| 1252 | 1 |
| 1253 | 1 |
| 1254 | 1 |
| 1255 | 1 |
| 1256 | 1 |
| 1257 | 1.6 |
| 1258 | 3 |
| 1259 | 4 |
| 1260 | 5 |
| 1261 | 6.3 |
| 1262 | 8 |
| 1263 | 10 |
| 1264 | 10.5 |
| 1265 | 9.5 |
| 1266 | 8.5 |
| 1267 | 7.6 |
| 1268 | 8.8 |
| 1269 | 11 |
| 1270 | 14 |
| 1271 | 17 |
| 1272 | 19.5 |
| 1273 | 21 |
| 1274 | 21.8 |
| 1275 | 22.2 |
| 1276 | 23 |
| 1277 | 23.6 |
| 1278 | 24.1 |
| 1279 | 24.5 |
| 1280 | 24.5 |
| 1281 | 24 |
| 1282 | 23.5 |
| 1283 | 23.5 |
| 1284 | 23.5 |
| 1285 | 23.5 |
| 1286 | 23.5 |
| 1287 | 23.5 |
| 1288 | 24 |
| 1289 | 24.1 |
| 1290 | 24.5 |
| 1291 | 24.7 |
| 1292 | 25 |
| 1293 | 25.4 |
| 1294 | 25.6 |
| 1295 | 25.7 |
| 1296 | 26 |
| 1297 | 26.2 |
| 1298 | 27 |
| 1299 | 27.8 |
| 1300 | 28.3 |
| 1301 | 29 |
| 1302 | 29.1 |
| 1303 | 29 |
| 1304 | 28 |
| 1305 | 24.7 |
| 1306 | 21.4 |
| 1307 | 18.1 |
| 1308 | 14.8 |
| 1309 | 11.5 |
| 1310 | 8.2 |
| 1311 | 4.9 |
| 1312 | 1.6 |
| 1313 | 0 |
| 1314 | 0 |
| 1315 | 0 |
| 1316 | 0 |
| 1317 | 0 |
| 1318 | 0 |
| 1319 | 0 |
| 1320 | 0 |
| 1321 | 0 |
| 1322 | 0 |
| 1323 | 0 |
| 1324 | 0 |
| 1325 | 0 |
| 1326 | 0 |
| 1327 | 0 |
| 1328 | 0 |
| 1329 | 0 |
| 1330 | 0 |
| 1331 | 0 |
| 1332 | 0 |
| 1333 | 0 |
| 1334 | 0 |
| 1335 | 0 |
| 1336 | 0 |
| 1337 | 0 |
| 1338 | 1.5 |
| 1339 | 4.8 |
| 1340 | 8.1 |
| 1341 | 11.4 |
| 1342 | 13.2 |
| 1343 | 15.1 |
| 1344 | 16.8 |
| 1345 | 18.3 |
| 1346 | 19.5 |
| 1347 | 20.3 |
| 1348 | 21.3 |
| 1349 | 21.9 |
| 1350 | 22.1 |
| 1351 | 22.4 |
| 1352 | 22 |
| 1353 | 21.6 |
| 1354 | 21.1 |
| 1355 | 20.5 |
| 1356 | 20 |
| 1357 | 19.6 |
| 1358 | 18.5 |
| 1359 | 17.5 |
| 1360 | 16.5 |
| 1361 | 15.5 |
| 1362 | 14 |
| 1363 | 11 |
| 1364 | 8 |
| 1365 | 5.2 |
| 1366 | 2.5 |
| 1367 | 0 |
| 1368 | 0 |
| 1369 | 0 |
| 1370 | 0 |
| 1371 | 0 |
| 1372 | 0 |
| 1373 | 0 |
| 1374 | 0 |
| 1375 | 0 |
| 1376 | 0 |
| 1377 | 0 |
| 1378 | 0 |
| 1379 | 0 |
| 1380 | 0 |
| 1381 | 0 |
| 1382 | 0 |
| 1383 | 0 |
| 1384 | 0 |
| 1385 | 0 |
| 1386 | 0 |
| 1387 | 0 |
| 1388 | 0 |
| 1389 | 0 |
| 1390 | 0 |
| 1391 | 0 |
| 1392 | 0 |
| 1393 | 0 |
| 1394 | 0 |
| 1395 | 0 |
| 1396 | 0 |
| 1397 | 0 |
| 1398 | 0 |
| 1399 | 0 |
| 1400 | 0 |
